# ﻿Review of Chinese species of the genus *Thoracostrongylus* Bernhauer, 1915 (Coleoptera, Staphylinidae, Staphylininae)

**DOI:** 10.3897/zookeys.1131.95038

**Published:** 2022-11-22

**Authors:** Mei-Hua Xia, Liang Tang, Harald Schillhammer

**Affiliations:** 1 College of Life Sciences, Shanghai Normal University, 100 Guilin Road, 1 Shanghai Normal University Shanghai China; 2 st Naturhistorisches Museum Wien Austria; 3 Educational Building 423 – A Room, Shanghai, 200234, China Shanghai Normal University Shanghai China; 4 Naturhistorisches Museum Wien, Burgring 7, A – 1010 Wien, Austria Naturhistorisches Museum Wien Austria

**Keywords:** Identification key, new records, new species, rove beetle, *Thoracostrongylus*

## Abstract

Species of the genus *Thoracostrongylus* Bernhauer, 1915 occurring in China are reviewed. Four new species and one new subspecies are described: *T.baishanzuensis***sp. nov.** (Zhejiang), *T.bicolor***sp. nov.** (Guangdong, Guangxi, Hunan, Yunnan), *T.brachypterus***sp. nov.** (Sichuan), *T.chrysites***sp. nov.** (Fujian), and *T.formosanusflavipes***ssp. nov**. (Zhejiang, Fujian, Hubei, Hunan, Sichuan, Guangxi, Guangdong, Anhui, Jiangxi). A new synonymy is proposed: *T.baoxingensis* Yang, Zhou & Schillhammer, 2011 **syn. nov.** is in fact *T.acerosus* Yang, Zhou & Schillhammer, 2011. New provincial records for *T.acerosus* Yang, Zhou & Schillhammer, 2011 are reported. A key to Chinese species of the genus is provided.

## ﻿Introduction

*Thoracostrongylus* Bernhauer, 1915 is a genus strictly distributed in east and southeast Asia. It was originally established as a subgenus of *Ontholestes* Ganglbauer, 1895, and later regarded as a separate genus (Blackwelder 1952). *Thoracostrongylus* can be readily distinguished from *Ontholestes* by the obtuse anterior angles of the pronotum (Smetana and Davies 2000), from *Lesonthotes* by the sparse, simple punctation of the forebody, and the sharply defined temples of the head (Brunke and Smetana 2019). Most species of *Thoracostrongylus* from China are very similar to each other in appearance. Recognition of some species is further complicated by the fact that the coloration is subject to a certain degree of variability. Dissection of male specimens should therefore be mandatory for identification of similar species. Additionally, the shape of the apex of the median lobe and paramere, which would normally be regarded as reliable characters for distinguishing species in related groups, is also variable in some species. Therefore, descriptions of new species in this genus should be based on very careful examination.

At present, sixteen species of the genus have been described worldwide, eleven of them recorded from China: *T.acerosus* Yang, Zhou & Schillhammer, 2011 from Hubei and Sichuan; *T.aduncatus* Yang, Zhou & Schillhammer, 2011 from Yunnan; *T.baoxingensis* Yang, Zhou & Schillhammer, 2011 from Sichuan; *T.birmanus* (Fauvel, 1895) from Hainan and Yunnan; *T.diaoluoensis* Yang, Zhou & Schillhammer, 2011 from Hainan; *T.formosanus* Shibata, 1982 from Zhejiang, Fujian, Hubei, Hunan, Sichuan and Taiwan; *T.fujianensis* Yang, Zhou & Schillhammer, 2011 from Fujian; *T.malaisei* Scheerpeltz, 1965 from Yunnan; *T.miyakei* Bernhauer, 1943 from Sichuan and Taiwan; *T.sarawakensis* (Bernhauer, 1915) from Hainan; and *T.velutinus* Scheerpeltz, 1965 from Yunnan. *Thoracostrongylusbaoxingensis* Yang, Zhou & Schillhammer, 2011 syn. nov. is here synonymized with *T.acerosus* Yang, Zhou & Schillhammer, 2011. The records of *T.formosanus* from mainland China, however, have turned out to be a distinct subspecies. Thus, including four new species described herein, the total number of *Thoracostrongylus* species is increased to 20 and the number of Chinese species is increased to 14 plus one subspecies.

## ﻿Materials and methods

The specimens examined in this paper were collected by sifting leaf litter, and by flight intercept traps and pitfall traps. They were subsequently killed with ethyl acetate. For examination of the genitalia, the last three abdominal segments were detached from the body after relaxing in hot water. The aedeagus together with other dissected pieces, were mounted in Euparal (Chroma Gesellschaft Schmidt, Koengen, Germany) on plastic slides beneath the card-mounted specimens. Photographs of sexual characters were taken with a Canon G9 camera attached to an Olympus SZX 16 stereoscope; habitus photographs were taken with a Canon macro lens MP-E 65 mm attached to a Canon EOS 7D camera and stacked with Zerene Stacker (http://www.zerenesystems.com/cms/stacker).

The specimens treated in this study are deposited in the following public and private collections:

**ASC** Aleš Smetana Collection, the National Museum of Nature and Science, Toshiba, Japan;

**BFC** Collection of Benedikt Feldmann, Münster, Germany;

**IZCAS**Institute of Zoology, Chinese Academy of Sciences, Beijing, P. R. China;

**MSC** Michael Schülke Collection, in Museum für Naturkunde, Berlin, Germany;

**NMW**Naturhistorisches Museum Wien, Austria;

**SHNU** Department of Biology, Shanghai Normal University, P. R. China;

**VAC** Volker Assing Collection, Hannover, Germany^†^ (will be deposited in Zoologisches Museum, Berlin).

Body measurements are abbreviated as follows:

**BL** body length, measured from the anterior margin of the clypeus to the posterior margin of abdominal tergite X;

**CL** length of eye;

**EL** length of elytra, measured from humeral angle;

**EW** width of elytra at the widest point;

**FL** forebody length, measured from the anterior margin of the clypeus to the apex of the elytra (apicolateral angle);

**HL** length of head along the midline;

**HW** width of head including eyes;

**PL** length of pronotum along the midline;

**PO** length of post-ocular region;

**PW** width of pronotum at the widest point.

## ﻿Taxonomic account

### ﻿ Thoracostrongylus acerosus

Taxon classificationAnimaliaColeopteraStaphylinidae

Yang, Zhou & Schillhammer, 2011

F57127A3-1163-56D6-B3CC-F8B8FDAB0E80

Figs 1–6 , 7–14 , 108 刺茎钝胸隐翅虫

Thoracostrongylus acerosus Yang, Zhou & Schillhammer, 2011: 410.
Thoracostrongylus baoxingensis Yang, Zhou & Schillhammer, 2011: 415. syn. nov.

#### Material examined.

China – **Sichuan Prov.** • 2♂♂, 1♀; Baoxing County, Fengtongzhai; 30°32'10"N, 102°54'20"E; alt. 1490 m; 22 July 2015; Jiang, Peng, Tu & Zhou leg.; SHNU • 1♂, 1♀; Baoxing County, Fengtongzhai N.R., Dengchigou; 30°32'N, 102°56'E; alt. 1870 m; 01 August 2016; Zhou, Jiang, Liu & Gao leg.; SHNU • 1♀; Baoxing County, Fengtongzhai N.R., Dengchigou; 30°29'N, 102°51'E; alt. 1692 m; 02 August 2016; Zhou, Jiang, Liu & Gao leg.; SHNU • 2♀♀; Tianquan County, Liangluxiang Village; 29°56'N, 102°23'E; alt. 1500–1700 m; 10 July 2012; Peng, Dai & Yin leg.; SHNU • 1♀; Tianquan County, Liangluxiang; 29°56'N, 102°23'E; alt. 1900–2000 m; Peng, Dai & Yin leg.; SHNU • 5♀♀; Tianquan County, Lianglu County; alt. 1400 m; 01 August 2011; Hao Huang leg.; SHNU • 2♂♂, 1♀; Dayi County, Xiling Snow Mt.; 30°38'6.25"N, 103°10'99.08"E; alt. 1250 m; 31 July 2021; Zhao & Cai leg.; SHNU. – **Shaanxi Prov.** • 1♀; Hanzhong, Tiantaishan; 33°16'20"N, 107°04'52"E; alt. 1326 m; 08 May 2021; Juan Li et al. leg.; SHNU • 2♀♀; Liuba, Huoshaodian; 33°30'08"N, 106°56'08"E; alt. 1041 m; 08 July 2021; Juan Li et al. leg.; SHNU • 2♀♀; Zhouzhi Coun., Houzhenzi, Qinling, west Sangongli Gou; 33°50'6.13"N, 107°48'52.4"E; alt. 1336 m; 17–19 May 2008; Huang & Xu leg.; SHNU • 1♀; Zhouzhi Coun., Houzhenzi, Qinling; 33°51'20.3"N, 107°50'18.3"E; alt. 1260 m; 05–10 May 2008; Huang & Xu leg.; SHNU • 1♂; Baoji City, Jiulongdong; 34°19'56"N, 106°52'22"E; alt. 986 m; 26 May 2021; Juan Li et al. leg.; SHNU • 1♀; Baoji City, Jiulongdong; 34°19'59"N, 106°52'21"E; alt. 975 m; 05 August 2021; Juan Li et al. leg.; SHNU • 1♀; Baoji City, Jiulongdong; 34°20'10"N, 106°51'51"E; alt. 969 m; 05 August 2021; Juan Li et al. leg.; SHNU • 1♂; Liuba, Zhangliang Temple; 33°41'51"N, 106°47'15"E; alt. 1476 m; 11 July 2021; Juan Li et al. leg.; SHNU • 1♀; Lueyang, Wulongdong; 33°31'16"N, 106°16'22"E; alt. 1107 m; 20 July 2021; Juan Li et al. leg.; SHNU • 1♀; Lueyang, Wulongdong; 33°30'51"N, 106°15'04"E; alt. 1237 m; 20 July 2021; Juan Li et al. leg.; SHNU • 2♂♂; Ankang City, Ningshan, Guanghuojie Town; 33°45'81"N, 108°46'48"E; alt. 1176 m; 07–08 May 2011; Bao-Xiang Zhan leg.; SHNU. – **Gansu Prov.** • 1♂; Hui County, Gaoqiaolinchang; 34°05'44"N, 105°57'42"E; alt. 1305 m; 18 May 2021; Juan Li et al. leg.; SHNU • 1♀; Hui County, Yanpinglinchang; 33°40'36"N, 106°16'51"E; alt. 1483 m; 18 July 2021; Juan Li et al. leg.; SHNU. – **Henan Prov.** • 1♂; Funiu Shan, Baotianman; alt. 1500–1700 m; 33°31'N, 111°56'E; 15 June 2009; J. Turna leg.; NMW.

**Figures 1–6.**
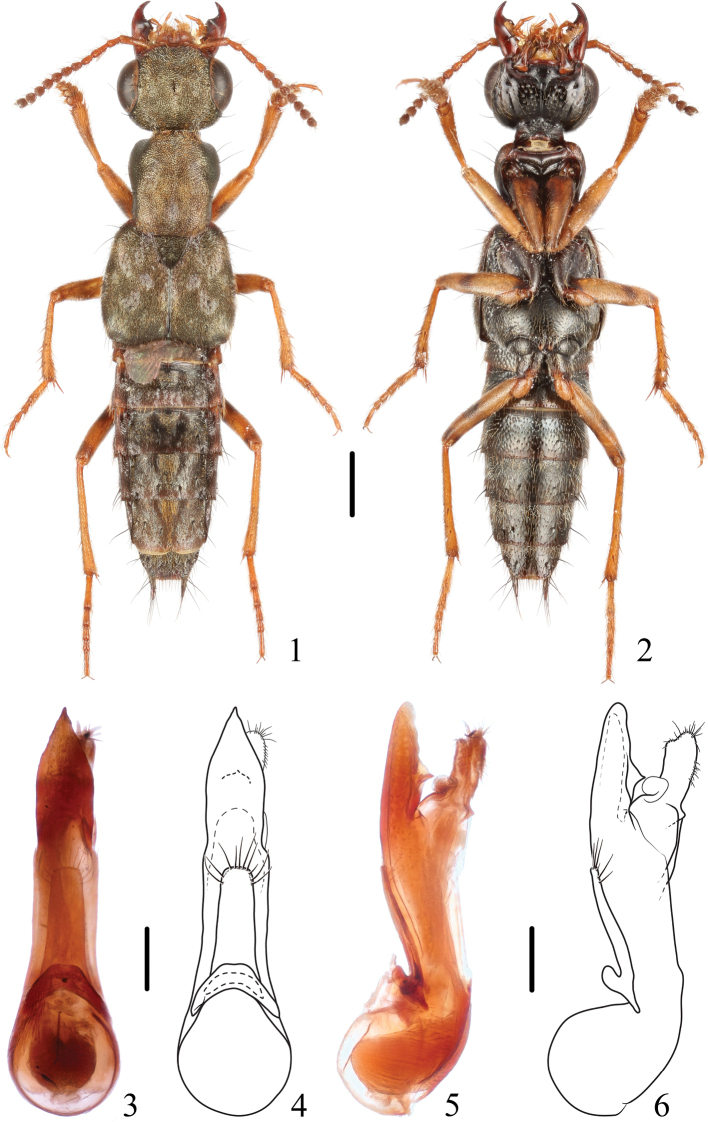
*Thoracostrongylusacerosus***1, 2** habitus **3–6** aedeagus, ventral (**3, 4**) and lateral (**5, 6**) views. Scale bars: 1 mm (**1, 2**); 0.2 mm (**3–6**).

#### Measurements.

**Male**: BL: 8.2–9.7 mm, FL: 4.5–5.2 mm. HL: 1.28–1.45 mm, HW: 1.78–1.95 mm, CL: 0.89–0.95 mm, PO: 0.22–0.28 mm, PL: 1.61–1.78 mm, PW: 1.45–1.50 mm, EL: 1.95–2.11 mm, EW: 1.95–2.11 mm. HL/HW: 0.70–0.78, CL/PO: 3.20–4.00, PL/PW: 1.12–1.23, EL/EW: 0.95–1.00, HW/EW: 0.87–0.94, PW/EW: 0.68–0.74, HW/PW: 1.23–1.31. **Female**: BL: 9.2–10.4 mm, FL: 4.8–5.3 mm. HL: 1.39–1.50 mm, HW: 2.00–2.17 mm, CL: 0.89–1.06 mm, PO: 0.22–0.28 mm, PL: 1.72–1.95 mm, PW: 1.50–1.72 mm, EL: 2.00–2.50 mm, EW: 2.11–2.50 mm. HL/HW: 0.68–0.72, CL/PO: 3.60–4.00, PL/PW: 1.00–1.19, EL/EW: 0.95–1.00, HW/EW: 0.84–0.95, PW/EW: 0.69–0.71, HW/PW: 1.23–1.33.

#### Distribution.

China (Hubei, Sichuan, Shaanxi, Gansu, Henan). New to Shaanxi, Gansu, and Henan.

#### Diagnosis.

In general appearance, *T.acerosus* is similar to *T.aduncatus* Yang, Zhou & Schillhammer, 2011, *T.fujianensis* Yang, Zhou & Schillhammer, 2011, and *T.diaoluoensis* Yang, Zhou & Schillhammer, 2011, but it can be recognized by the sharply pointed tip of aedeagal median lobe.

#### Remarks.

The apical portion of the median lobe and the paramere are subject to some variability (Figs 3–14). This may be observed not only in populations from different localities but also within one population. A closer inspection of the types of *T.baoxingensis* and *T.acerosus* revealed that this is the case here as well and that both species are conspecific.

**Figures 7–14.**
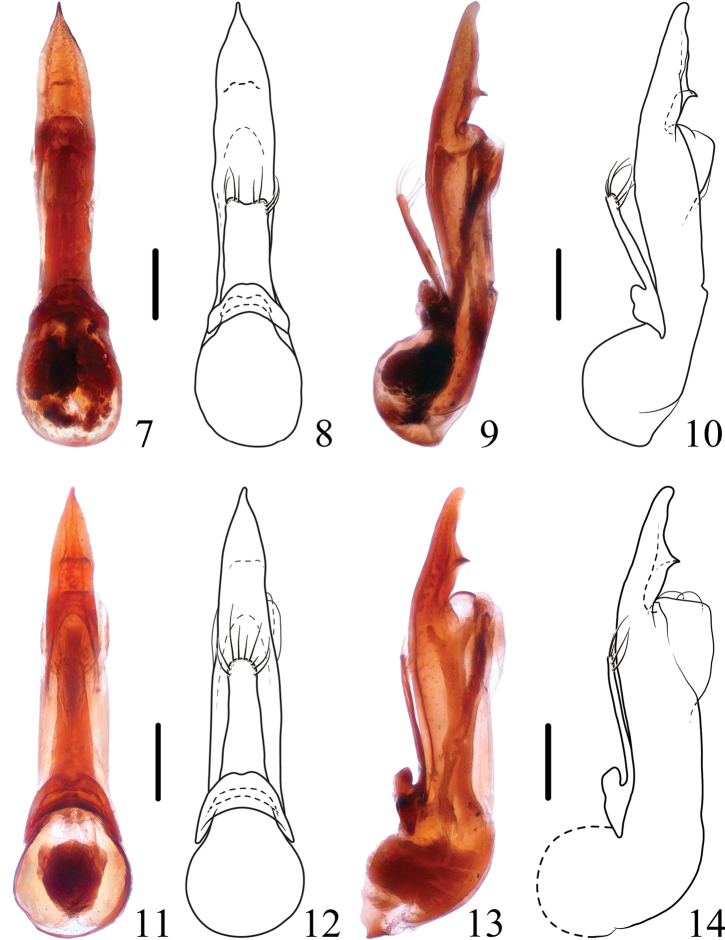
*Thoracostrongylusacerosus***7–10** aedeagus from Xiling Snow Mountain, ventral (**7, 8**) and lateral (**9, 10**) views **11–14** aedeagus from Jiulongdong, ventral (**11, 12**) and lateral (**13, 14**) views. Scale bars: 0.2 mm.

### ﻿ Thoracostrongylus aduncatus

Taxon classificationAnimaliaColeopteraStaphylinidae

Yang, Zhou & Schillhammer, 2011

32382852-59A7-5375-96B7-3DE45E4021DD

Figs 15–20 , 21–28 , 29–32 , 109 钩茎钝胸隐翅虫

Thoracostrongylus aduncatus Yang, Zhou & Schillhammer, 2011: 413.

#### Material examined.

China – **Yunnan Prov.** • 1♂; Xishuangbanna, Menglong Town, Mengsong; 20°30'41"N, 100°30'19"E; alt. 1700 m; 03 April 2018; Peng, Shen & Cheng leg.; SHNU • 1♂; Nabanhe N.R., Chuguohe, Bengganghani; alt. 1750 m; 28 April 2009; Hu & Yin leg.; SHNU • 1♂; Nabanhe N.R., Bengganghani, Nanmugaha; alt. 1650 m; 30 April 2009; Hu & Yin leg.; SHNU • 1♂, 1♀; Nabanhe N.R., Shanshenmiao, Bengganghani; alt. 1700 m; 27 April 2009; Hu & Yin leg.; SHNU • 2♀♀; Nabanhe N.R., Bengganghani; alt. 1750 m; 03 May 2009; Hu & Yin leg.; SHNU • 4♂♂, 1♀; Baoshan City, Mangkuan Town, Baihualing; 25°18'11"N, 98°47'38"E; alt. 1900 m; 21 April 2013; Dai, Peng & Song leg.; SHNU.

**Figures 15–20.**
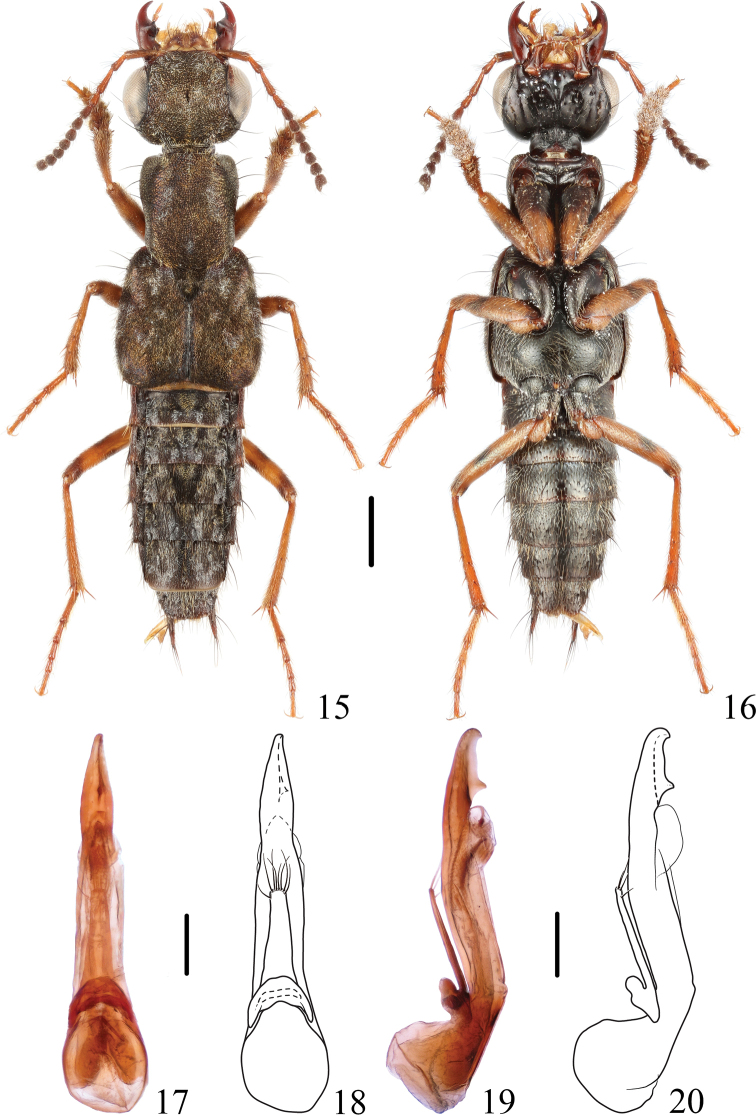
*Thoracostrongylusaduncatus***15, 16** habitus **17–20** aedeagus, ventral (**17, 18**) and lateral (**19, 20**) views. Scale bars: 1 mm (**15, 16**); 0.2 mm (**17–20**).

#### Measurements.

**Male**: BL: 7.0–8.3 mm, FL: 4.2–5.1 mm. HL: 1.17–1.39 mm, HW: 1.61–1.89 mm, CL: 0.83–0.95 mm, PO: 0.17–0.28 mm, PL: 1.50–1.78 mm, PW: 1.22–1.50 mm, EL: 1.78–2.11 mm, EW: 1.78–2.11 mm. HL/HW: 0.70–0.77, CL/PO: 3.00–5.00, PL/PW: 1.12–1.27, EL/EW: 0.97–1.00, HW/EW: 0.84–0.91, PW/EW: 0.66–0.71, HW/PW: 1.24–1.32. **Female**: BL: 8.0–9.6 mm, FL: 4.8–5.3 mm. HL: 1.33–1.50 mm, HW: 1.83–2.06 mm, CL: 0.95–1.00 mm, PO: 0.22–0.28 mm, PL: 1.72–1.83 mm, PW: 1.39–1.56 mm, EL: 2.00–2.11 mm, EW: 2.11–2.22 mm. HL/HW: 0.73, CL/PO: 3.60–4.25, PL/PW: 1.18–1.24, EL/EW: 0.95, HW/EW: 0.87–0.93, PW/EW: 0.66–0.70, HW/PW: 1.32.

**Figures 21–28.**
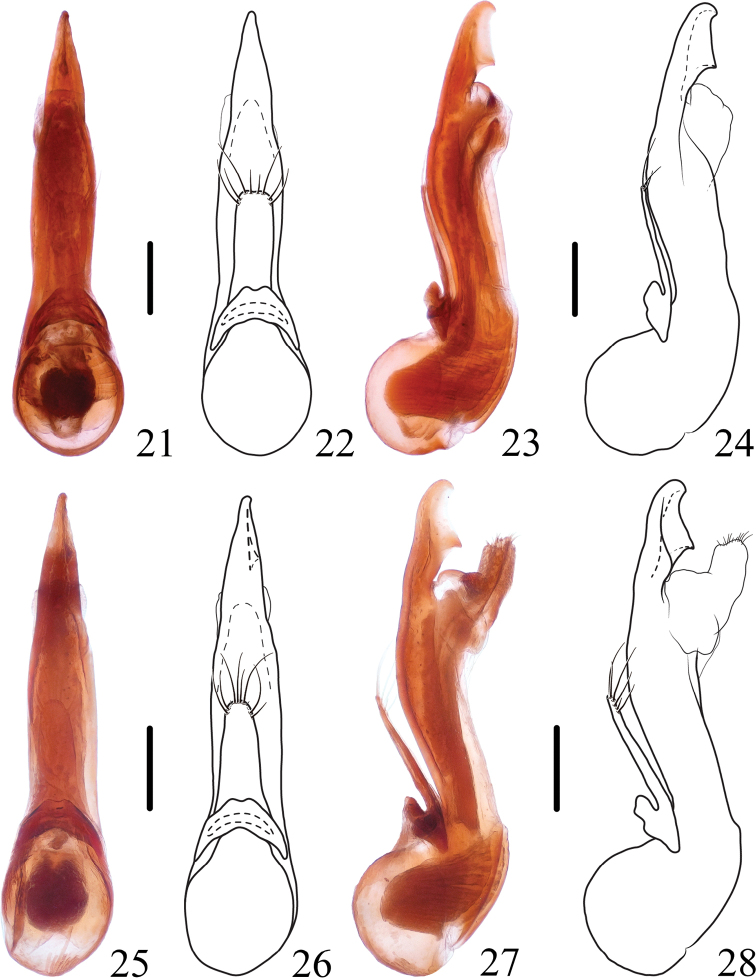
*Thoracostrongylusaduncatus***21–24** aedeagus from Baihualing, ventral (**21, 22**) and lateral (**23, 24**) views **25–28** aedeagus from Baihualing, ventral (**25, 26**) and lateral (**27, 28**) views. Scale bars: 0.2 mm.

#### Diagnosis.

The species is similar to *T.acerosus* Yang, Zhou & Schillhammer, 2011, *T.fujianensis* Yang, Zhou & Schillhammer, 2011, and *T.diaoluoensis* Yang, Zhou & Schillhammer, 2011 in general appearance, but it can be distinguished from them by the apex of median lobe pointed dorsad forming an apical tooth in lateral view, and from *T.diaoluoensis* also by the aedeagal median lobe with a subapical tooth on the dorsal side. Aedeagal variation (Figs 17–32) occurs in the apical parts of median lobe and paramere.

**Figures 29–32.**
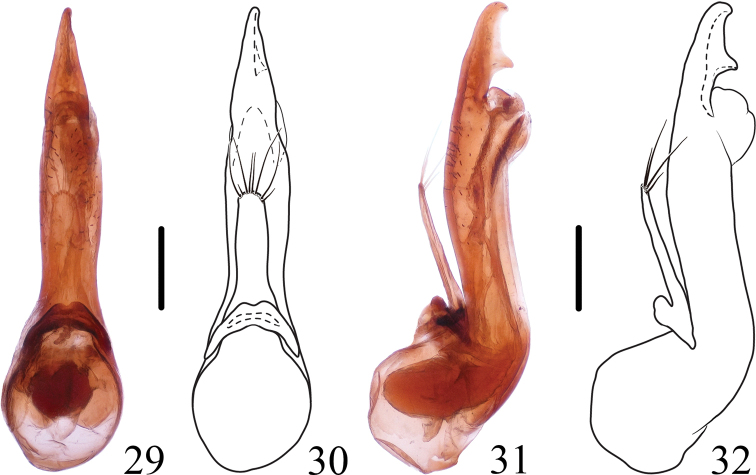
*Thoracostrongylusaduncatus***29–32** aedeagus from Nabanhe, ventral (**29, 30**) and lateral (**31, 32**) views. Scale bars: 0.2 mm.

#### Distribution.

China (Yunnan).

### ﻿ Thoracostrongylus birmanus

Taxon classificationAnimaliaColeopteraStaphylinidae

(Fauvel, 1895)

9FE3F7F4-9C03-5B98-97E2-29815DE8B0A3

Figs 33–39 , 110 缅甸钝胸隐翅虫

Leistotrophus birmanus Fauvel, 1895: 246.
Ontholestes birmanus : Bernhauer & Schubert, 1914: 392.
Thoracostrongylus birmanus : Cameron, 1932: 214; Yang et al. 2011: 422.

#### Material examined.

China – **Yunnan Prov.** • 1♂; Xishuangbanna, Nabanhe N.R.; 18 June 2009; Ling-Zeng Meng leg.; SHNU • 1♂; Nabanhe N.R., Manfei; 22°09'30.5"N, 100°41'29.1"E; alt. 620 m; 18 November 2008; Hu & Tang leg.; SHNU • 1♂, 1♀; Nabanhe Conv., Manfei; 10 January 2004; Li & Tang leg.; SHNU • 1♂, 1♀; Nabanhe Conv., Manfei; 09 January 2004; Li & Tang leg.; SHNU • 1♀; Mengla County, Menglun Town; alt. 550 m; 26 April 2014; Jian-Yue Qiu leg.; SHNU • 2♂♂; Baoshan City, Baihualing; 25°17'39"N, 98°48'09"E; alt. 1350–1450 m; 19 April 2013; Song, Peng & Dai leg.; SHNU • 1♂, 1♀; Xishuangbanna, Jinghong City; 22°02'19"N, 100°55'23"E; alt. 1000–1080 m; 29 November 2016; Jiang, Liu, Huang & Liu leg.; SHNU • 1♂; Lincang, Shuibatou Village; 24°38'16"N, 100°29'17"E; alt. 1281 m; 20 June 2019; Zi-Chun Xiong leg.; SHNU • 1♀; Baoshan, Longyang baihualing; 25°20'35"N, 98°49'01"E; alt. 1400–1900 m; 20–23 June 2020; Lu Qiu leg.; SHNU. – **Hainan Prov.** • 2♂♂, 2♀♀; Ledong County, Jianfengling, Mingfenggu; 18°44'43"N, 108°50'20"E; alt. 956–1048 m; 20–21 April 2018; Ri-Xin Jiang leg.; SHNU • 1♂, 1♀; Wuzhishan City, Mt. Wuzhishan; 18°54'N, 109°41'E; alt. 650–700 m; 20 April 2012; Peng & Dai leg.; SHNU • 1♂; Ledong County, Jianfengling; alt. 950 m; 15 April 2010; Jian-Qing Zhu leg.; SHNU • 2♀♀; Ledong County, Jianfengling N.R.; alt. 910 m; 15 April 2010; Ting Feng leg.; SHNU • 1♂; Wuzhishan Mt., Guanshandian; 18°53'N, 109°41'E; alt. 650 m; 19 April 2012; Pan & Li leg.; SHNU • 1♀; Changjiang County, Bawangling; alt. 1000 m; 14 November 2006; Li-Zhen Li leg.; SHNU • 1♀; Baoshan County, Maoganxiang; 14 April 2015; Lu Qiu leg.; SHNU • 1♀; Qiongzhong County, Limu Mt., N.R.; 19°10'04"N, 109°44'45"E; alt. 625 m; 29 January 2015; Peng, Yin, Tu, Song, Shen, Zhou, Yan & Wang leg.; SHNU.

**Figures 33–39.**
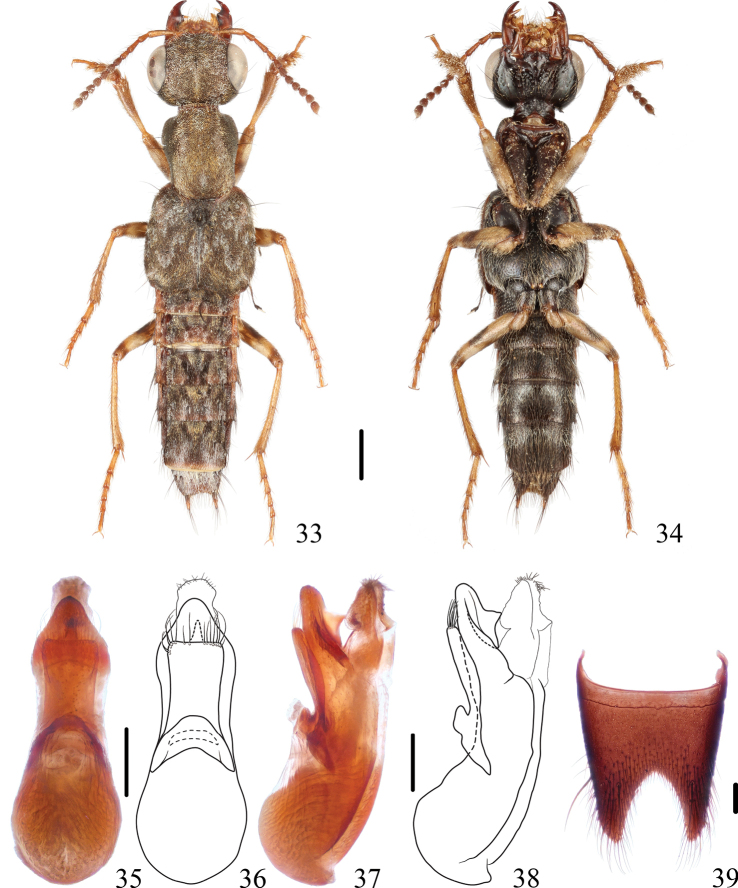
*Thoracostrongylusbirmanus***33, 34** habitus **35–38** aedeagus, ventral (**35, 36**) and lateral (**37, 38**) views **39** male abdominal sternite VIII. Scale bars: 1 mm (**33, 34**); 0.2 mm (**35–39**).

#### Measurements.

**Male**: BL: 6.8–9.1 mm, FL: 4.4–5.2 mm. HL: 1.33–1.50 mm, HW: 1.78–2.06 mm, CL: 0.89–1.00 mm, PO: 0.28 mm, PL: 1.56–1.78 mm, PW: 1.39–1.61 mm, EL: 1.83–2.17 mm, EW: 1.95–2.22 mm. HL/HW: 0.70–0.75, CL/PO: 3.20–3.60, PL/PW: 1.10–1.19, EL/EW: 0.92–0.98, HW/EW: 0.90–0.93, PW/EW: 0.68–0.74, HW/PW: 1.24–1.37. **Female**: BL: 7.5–10.8 mm, FL: 4.8–5.8 mm. HL: 1.39–1.67 mm, HW: 1.89–2.22 mm, CL: 0.95–1.11 mm, PO: 0.22–0.28 mm, PL: 1.72–2.00 mm, PW: 1.50–1.67 mm, EL: 1.95–2.39 mm, EW: 2.11–2.45 mm. HL/HW: 0.72–0.75, CL/PO: 3.40–4.50, PL/PW: 1.15–1.21, EL/EW: 0.92–0.98, HW/EW: 0.84–0.91, PW/EW: 0.66–0.71, HW/PW: 1.24–1.33.

#### Diagnosis.

The species may be easily recognized by the combination of following characters: abdominal sternites with longer and denser pubescence, male sternite VIII (Fig. 39) with deep medio-apical emargination, and male sternite VII slightly emarginate medio-apically.

#### Distribution.

China (Yunnan, Hainan), India, and Myanmar.

### ﻿ Thoracostrongylus diaoluoensis

Taxon classificationAnimaliaColeopteraStaphylinidae

Yang, Zhou & Schillhammer, 2011

03AED02D-FB97-5B4D-B453-1ADDB3911A03

**Figures 40–45.**
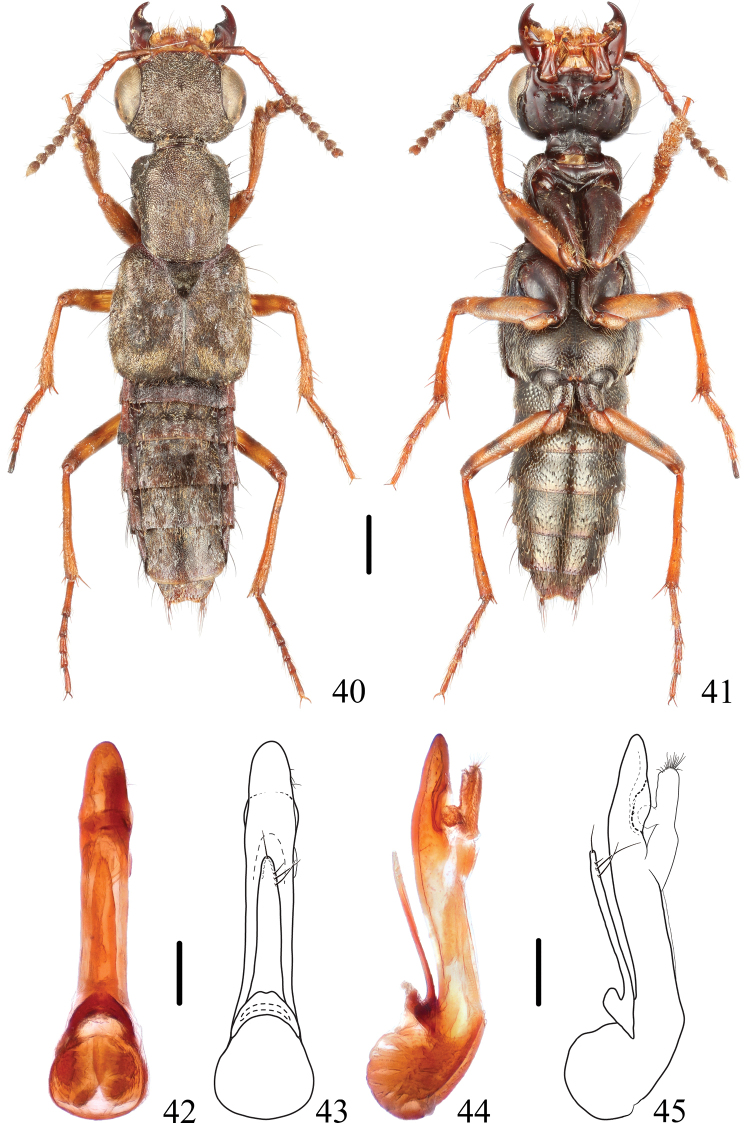
*Thoracostrongylusdiaoluoensis***40, 41** habitus **42–45** aedeagus, ventral (**42, 43**) and lateral (**44, 45**) views. Scale bars: 1 mm (**40, 41**); 0.2 mm (**42–45**).

Figs 40–45 , 111 吊罗钝胸隐翅虫

Thoracostrongylus diaoluoensis Yang, Zhou & Schillhammer, 2011: 418.

#### Material examined.

China – **Hainan Prov.** • 1♂; Changjiang County, Bawangling; alt. 1000 m; 14 November 2006; Li-Zhen Li leg.; SHNU.

#### Measurements.

**Male**: BL: 9.2 mm, FL: 5.6 mm. HL: 1.56 mm, HW: 2.28 mm, CL: 1.06 mm, PO: 0.28 mm, PL: 2.06 mm, PW: 1.72 mm, EL: 2.39 mm, EW: 2.39 mm. HL/HW: 0.68, CL/PO: 3.80, PL/PW: 1.19, EL/EW: 1.00, HW/EW: 0.95, PW/EW: 0.72, HW/PW: 1.32.

#### Diagnosis.

The apical portion of the median lobe (Figs 42–45) of the specimen examined here is a little wider than that of the type illustrated in the original description, which is considered as intraspecific variation. The species can be recognized from similar species by median lobe of the aedeagus without an apical or subapical tooth on the dorsal side.

#### Distribution.

China (Hainan).

### ﻿ Thoracostrongylus formosanus formosanus

Taxon classificationAnimaliaColeopteraStaphylinidae

Shibata, 1982

3F3025A9-2C1F-5AE7-B9DB-D1778FD05B77

Fig. 112 台湾钝胸隐翅虫指名亚种

Thoracostrongylus formosanus Shibata, 1982: 71; Yang et al. 2011: 424; Hu, 2020: 348.

#### Material examined.

China – **Taiwan Prov.** • 10 exs.; Hualien, Guanyuan; 24°11'12"N, 121°20'00"E; alt. 2200–2300 m; 27 June 2006; Y.-F. Hsu leg.; NMW • 16 exs.; Hualien, Pilu; alt. 2100 m; 24°10'58"N, 121°23'16"E; 06 May 2006; Y.-F. Hsu leg.; NMW.

#### Measurements.

BL: 8.5–10.5 mm, FL: 5.0–5.8 mm. HL: 1.25–1.60 mm, HW: 1.8–2.2 mm, CL: 0.85–0.85 mm, PO: 0.3–0.4 mm, PL: 1.75–2.00 mm, PW: 1.5–1.7 mm, EL: 2.1–2.5 mm, EW: 2.20–2.65 mm. HL/HW: 0.69–0.73, CL/PO: 2.38–2.86, PL/PW: 1.16–1.17, EL/EW: 0.94–0.95, HW/EW: 0.81–0.83.

#### Diagnosis.

The subspecies is most similar to *T.velutinus* from Yunnan and Myanmar, but can be easily distinguished by the usually black mid and hind tibiae and tarsi. Both differ from other species from east and southeast China in the abdominal tergites III–VI without triangular mediobasal golden tomentose patch.

#### Distribution.

China (Taiwan).

### ﻿ Thoracostrongylus formosanus flavipes ssp.nov.

Taxon classificationAnimaliaColeopteraStaphylinidae

866A88F9-E32C-5EB6-A6C9-BD751425F968

https://zoobank.org/811E5361-8C24-41BE-8BB2-5C2AF207A456

Figs 46–52 , 113 台湾钝胸隐翅虫黄足亚种

#### Type material.

***Holotype.*** China – **Zhejiang Prov.** • ♂, glued on a card with labels as follows: “China: Zhejiang, Longquan, Fengyang Mt., Guanyintai; alt. 1000 m; 11 May 2019; Tang & Zhao leg.” “Holotype / *Thoracostrongylus formosanus flavipes* / Xia, Tang & Schillhammer” [red handwritten label]; SHNU. ***Paratypes***. China – **Zhejiang Prov.** • 4♂♂, 6♀♀; same data as holotype; SHNU • 1♂; Longquan City, Fengyangshan, Lu’ao Vill.; 27°55'8.95"N, 119°11'55.54"E; alt. 1200–1300 m; 16–17 July 2018; Zi-Wei Yin leg.; SHNU • 2♂♂, 1♀; Longquan City, Fengyangshan N.R., Lu’aocun Village; 27°55'19.66"N, 119°11'38.86"E; alt. 1076 m; 04 May 2016; Jiang, Liu & Zhou leg.; SHNU • 1♂; Longquan City, Fengyangshan N.R., Datianping; 27°54'29.67"N, 119°10'31.45"E; alt. 1350 m; 30 April 2016; Jiang, Liu & Zhou leg.; SHNU • 2♀♀; Longquan City, Fengyangshan N.R., Datianping; 27°54'29.67"N, 119°10'31.45"E; alt. 1350 m; 30 April 2016; Jiang, Liu & Zhou leg.; SHNU • 2♂♂, 1♀; Jinhua City, Pan’an County, Dapanshan N.R.; 28°58'41.03"N, 120°29'19.24"E; alt. 531–783 m; 08 May 2016; Jiang, Liu & Zhou leg.; SHNU • 1♂, 2♀♀; Lishui City, Qingyuan County, Baishanzu, Station to Peak; 27°45'20"N, 119°11'78"E; alt. 1721 m; 24 April 2015; Song & Yan leg.; SHNU • 2♀♀; Longquan City, Fengyangshan N.R., Mihougu, near stream; 27°55'0.18"N, 119°11'52.91"E; alt. 1116 m; 03 May 2016; Jiang, Liu & Zhou leg.; SHNU • 1♀; Wuyanling; alt. 700 m; 09 May 2004; Hu, Tang & Zhou leg.; SHNU. – **Guangxi Prov.** • 7♂♂, 16♀♀; Huanjiang, Jiuwan Mt., Yangmeiao; 25°12'22.15"N, 108°40'32.01"E; alt. 1250 m; 25 April 2021; Tang, Peng, Cai & Song leg.; SHNU • 1♂; Huanjiang, Jiuwan Mt., Yangmeiao; 25°12'22.15"N, 108°40'32.01"E; alt. 1250 m; 08 May 2021; Tang, Peng, Cai & Song leg.; SHNU • 1♀; Huanjiang, Jiuwan Mt., Yangmeiao; 25°12'22.15"N, 108°40'32.01"E; alt. 1250 m; 23 April 2021; Tang, Peng, Cai & Song leg.; SHNU • 1♂; Jinxiu County, Mt. Shengtangshan; alt. 1500 m; 26 July 2011; Zhong Peng leg.; SHNU • 1♀; Guilin City, Huaping N.R., Anjiangping; alt. 1500 m; 18 July 2011; Liang Tang leg.; SHNU. – **Guangdong Prov.** • 4♂♂, 2♀♀; Ruyuan County, Nanling N.R., Qingshui Valley; 24°54'57"N, 113°01'55"E; alt. 900 m; 04 May 2015; Peng, Tu & Zhou leg.; SHNU • 1♂, 2♀♀; Ruyuan County, Nanling N.R., Laopengkeng; 24°56'29"N, 113°00'27"E; alt. 1360 m; 29 April 2015; Peng, Tu & Zhou leg.; SHNU • 6♂♂, 3♀♀; Ruyuan County, Nanling N.R., Baobaoshan Station; 24°55'43"N, 113°00'58"E; alt. 1030 m; 25 April 2015; Peng, Tu & Zhou leg.; SHNU. – **Sichuan Prov.** • 3♂♂, 1♀; Dayi County, Xiling Snow Mt.; 30°38'6.25"N, 103°10'99.08"E; alt. 1250 m; 31 July 2021; Zhao & Cai leg.; SHNU • 1♀; Jiulong County, Hongba; alt. 2000 m; 13 August 2005; Ming Yi leg.; SHNU. – **Anhui Prov.** • 1♀; Huangshan, Tangkou Town, Hougu; 30°05'3.48"N, 118°08'45.96"E; alt. 569–688 m; 29 June–03 July 2020; Chong Li leg.; SHNU. – **Jiangxi Prov.** • 1♂, 1♀; Yichun City, Fengxin County, Baizhang Vill.; 28°42'55"N, 114°46'14"E; alt. 1000–1300 m; 16 July 2013; Hu & Lv leg.; SHNU • 1♂; Longnan County, Jiulianshan, summit of Huangniushi; 24°30'53"N, 114°26'6.72"E; alt. 1000–1230 m; 12 May 2021; Zhou & Li leg.; SHNU • 1♂; Ji’an City, Jinggangshan, Huangyangjie; 26°37'25"N, 114°06'58"E; alt. 1240 m; 28.vii,2014; Chen, Hu, Lv & Yu leg.; SHNU • 1♀; Pingxiang City, Gaozhou County, Gaotianyan; 27°23'51"N, 114°00'54"E; alt. 1025 m; 23 July 2013; Song, Yin & Yu leg.; SHNU. – **Hunan Prov.** • 2♂♂; Liuyang City, Daweishan; 28°25'25"N, 114°05'57"E; alt. 1300 m; 06 June 2014; Peng, Shen, Yu & Yan leg.; SHNU • 2♂♂; Liuyang City, Daweishan, 28°25'25"N, 114°05'57"E; alt. 1300 m, 07 June 2014; Peng, Shen, Yu & Yan leg.; SHNU • 2♂♂, 1♀; Yanling County, Nanfengmian; 26°18'N, 114°01'E; alt. 1855 m; 07 June 2015; Peng, Shen, Tu & Zhou leg.; SHNU • 1♂; Xin’ning County, Shunhuang Mt., Yangheping; 26°23'41.58"N, 111°00'08.16"E; alt. 820 m; 02 May 2021; Yin, Zhang, Pan & Shen leg.; SHNU • 1♂; Xin’ning County, Shunhuang Mt., Yangheping; 26°23'41.58"N, 111°00'08.16"E; alt. 820 m; 30 April 2021; Yin, Zhang, Pan & Shen leg.; SHNU • 1♂; Chengzhou, Yizhang County, Mangshan N.R.; 24°56'26"N, 112°59'18"E; alt. 1400 m; 26 April 2015; Peng, Tu & Zhou leg.; SHNU • 2♀♀; Liuyang City, Daweishan; 28°25'N, 114°05'E; alt. 1000 m; 11 June 2015; Peng, Shen, Tu & Zhou leg.; SHNU • 2♂♂, 3♀♀; Mangshan N.R.; 10 May 2020; SHNU • 6♂♂, 12♀♀; Yanling County, Nanfengmian; 26°18'10"N, 114°00'12"E; alt. 1620 m; 26 May 2014; Peng, Shen, Yu & Yan leg.; SHNU • 1♂, 1♀; Yanling County, Nanfengmian; 26°16'32"N, 113°59'34"E; alt. 1380 m; 27 May 2014; Peng, Shen, Yu & Yan leg.; SHNU • 2♂♂, 1♀; Yanling County, Nanfengmian; 26°18'20"N, 114°00'51"E; alt. 1730 m; 28 May 2014; Peng, Shen, Yu & Yan leg.; SHNU. – **Fujian Prov.** • 15♂♂, 14♀♀; Wuyishan City, Guadun Vill.; 27°44'N, 117°38'E; alt. 1300–1500 m; 27 May 2012; Peng & Dai leg.; SHNU • 2♂♂, 1♀; Wuyishan City, Guadun Vill.; 27°44'N, 117°38'E; alt. 1200–1500 m; 26 May 2012; Peng & Dai leg.; SHNU • 2♂♂, 2♀♀; Wuyishan City, Guadun Vill.; 27°44'N, 117°38'E; alt. 1200–1500 m; 25 May 2012; Peng & Dai leg.; SHNU • 3♀♀; Wuyishan City, Guadun Vill.; 27°44'N, 117°38'E; alt. 1200–1300 m; 24 May 2012; Peng & Dai leg.; SHNU • 1♀; Wuyishan City, Guadun Vill.; 27°44'N, 117°38'E; alt. 1100–1400 m; 29 May 2012; Peng & Dai leg.; SHNU • 1♂, 2♀♀; Wuyishan City, Guadun Vill.; 27°44'N, 117°38'E; alt. 1800 m; 01 June 2012; Peng & Dai leg.; SHNU • 1♂; Guadun Vill.; August 2008; Zhu-Qing He leg.; SHNU • 2♂♂, 1♀; Guihe Vill., Meihua Mt.; alt. 1500 m; 20 May 2007; Huang & Xu leg.; SHNU • 1♂; N. Slope Gouzinao, Meihua Mt.; alt. 1650 m; 29 May 2007; Huang & Xu leg.; SHNU • 1♂; Guihe Vill., Gouzinao, Meihua Mt.; alt. 1500 m; 26 May 2007; Huang & Xu leg.; SHNU.

**Figures 46–52.**
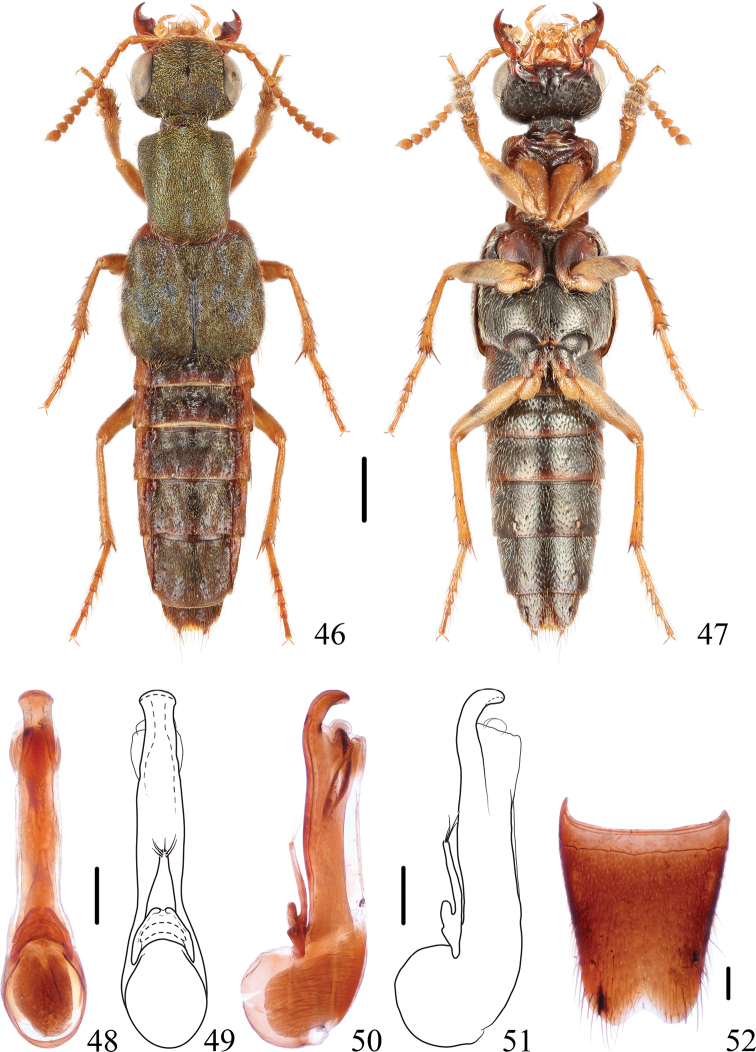
*Thoracostrongylusformosanusflavipes* ssp. nov. **46, 47** habitus **48–51** aedeagus, ventral (**48, 49**) and lateral (**50, 51**) views **52** male abdominal sternite VIII. Scale bars: 1 mm (**46, 47**); 0.2 mm (**48–52**).

#### Measurements.

**Male**: BL: 9.0–10.8 mm, FL: 5.0–5.8 mm. HL: 1.22–1.56 mm, HW: 1.72–2.11 mm, CL: 0.89–1.06 mm, PO: 0.22–0.28 mm, PL: 1.78–2.00 mm, PW: 1.45–1.72 mm, EL: 2.11–2.45 mm, EW: 2.17–2.56 mm. HL/HW: 0.69–0.76, CL/PO: 3.20–4.00, PL/PW: 1.16–1.23, EL/EW: 0.95–0.97, HW/EW: 0.79–0.83, PW/EW: 0.66–0.67, HW/PW: 1.18–1.23. **Female**: BL: 8.2–11.7 mm, FL: 4.7–5.6 mm. HL: 1.22–1.45 mm, HW: 1.61–2.00 mm, CL: 0.83–0.89 mm, PO: 0.22–0.28 mm, PL: 1.67–2.00 mm, PW: 1.33–1.67 mm, EL: 2.00–2.56 mm, EW: 2.00–2.67 mm. HL/HW: 0.70–0.76, CL/PO: 3.00–3.75, PL/PW: 1.20–1.25, EL/EW: 0.95–1.00, HW/EW: 0.75–0.81, PW/EW: 0.63–0.68, HW/PW: 1.18–1.21.

#### Diagnosis.

The new subspecies differs from the nominate subspecies in the slightly shorter tempora, and entirely reddish to yellowish antennae and legs (except a dark band on the femora), while the nominate subspecies has almost entirely dark antennae, and black tibiae and tarsi. Even in paler (teneral) specimens of the nominate subspecies, the antennae and legs are at least partly darkened.

#### Distribution.

The subspecies is widespread in China (Zhejiang, Fujian, Hubei, Hunan, Sichuan, Guangxi, Guangdong, Anhui, Jiangxi).

### ﻿ Thoracostrongylus fujianensis

Taxon classificationAnimaliaColeopteraStaphylinidae

Yang, Zhou & Schillhammer, 2011

DA43D8B5-9634-5424-A156-9BC1A3BD6BE3

Figs 53–58 , 59–66 , 67–70 , 114 福建钝胸隐翅虫

Thoracostrongylus fujianensis Yang, Zhou & Schillhammer, 2011: 419.

#### Material examined.

China – **Fujian Prov.** • 1♂, 1♀; Wuyishan City, Guadun Vill.; 27°44'N, 117°38'E; alt. 1200–1500 m; 25 May 2012; Peng & Dai leg. (SHNU) • 3♂♂, 1♀; Wuyishan City, Guadun Vill.; 27°44'N, 117°38'E; alt. 1200–1500 m; 26 May 2012; Peng & Dai leg.; SHNU • 1♂; Wuyishan City, Guadun Vill.; 27°44'N, 117°37'E; alt. 1200–1500 m; 28 May 2012; Peng & Dai leg.; SHNU • 1♂; Wuyishan City, Guadun Vill.; 27°44'N, 117°38'E; alt. 1300–1500 m; 27 May 2012; Peng & Dai leg.; SHNU • 1♂; Wuyishan City, Guadun Vill.; 27°44'N, 117°38'E; alt. 1100–1300 m; 30 May 2012; Peng & Dai leg.; SHNU • 2♂♂; Wuyishan City, Guadun Vill.; 27°44'N, 117°38'E; alt. 1300 m; 02 June 2012; Peng & Dai leg.; SHNU • 3♀♀; Guadun; August 2008; Zhu-Qing He leg.; SHNU • 2♀♀; Wuyishan, Guadun; alt. 1200 m; 30 August 2009; Hao Huang leg.; SHNU • 1♂; Mt. Wuyi; 27–31 May 2012; Li-Zhen Li leg.; SHNU • 1♂; Longkeng Vill., Junzifeng; alt. 1400 m; 07 August 2008; Qi & Yin leg.; SHNU • 1♂; Guihe Vill., Meihua Mt.; alt. 1500 m; 27 May 2007; Huang & Xu leg.; SHNU • 1♂; Guihe Vill., Meihua Mt.; alt. 1500 m; 20 May 2007; Huang & Xu leg.; SHNU.

**Figures 53–58.**
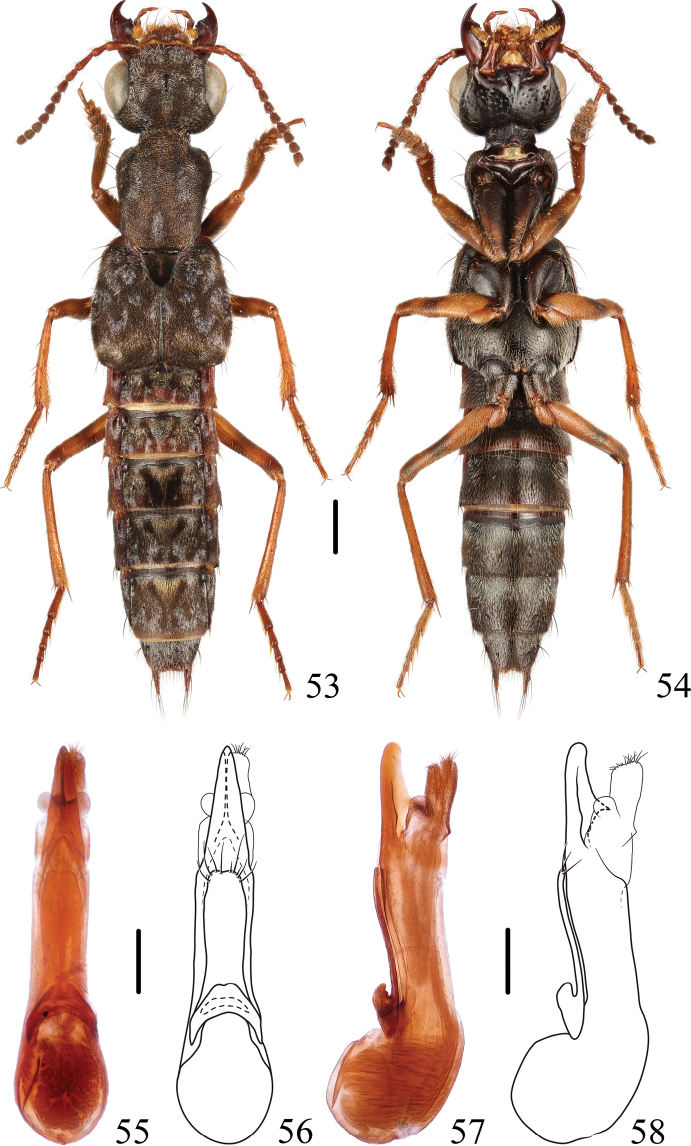
*Thoracostrongylusfujianensis***53, 54** habitus **55–58** aedeagus, ventral (**55, 56**) and lateral (**57, 58**) views. Scale bars: 1 mm (**53, 54**); 0.2 mm (**55–58**).

#### Measurements.

**Male**: BL: 7.7–11.1 mm, FL: 4.4–5.8 mm. HL: 1.22–1.56 mm, HW: 1.72–2.17 mm, CL: 0.83–1.00 mm, PO: 0.22–0.28 mm, PL: 1.50–2.06 mm, PW: 1.33–1.72 mm, EL: 1.83–2.34 mm, EW: 1.83–2.39 mm. HL/HW: 0.70–0.76, CL/PO: 3.00–4.50, PL/PW: 1.13–1.21, EL/EW: 0.95–1.00, HW/EW: 0.85–0.95, PW/EW: 0.69–0.75, HW/PW: 1.22–1.32. **Female**: BL: 8.8–10.3 mm, FL: 5.1–5.6 mm. HL: 1.39–1.61 mm, HW: 1.95–2.22 mm, CL: 0.95–1.11 mm, PO: 0.22–0.28 mm, PL: 1.83–2.06 mm, PW: 1.50–1.72 mm, EL: 2.06–2.39 mm, EW: 2.11–2.45 mm. HL/HW: 0.69–0.74, CL/PO: 3.40–4.25, PL/PW: 1.17–1.22, EL/EW: 0.95–1.00, HW/EW: 0.90–0.95, PW/EW: 0.70–0.72, HW/PW: 1.27–1.33.

**Figures 59–66.**
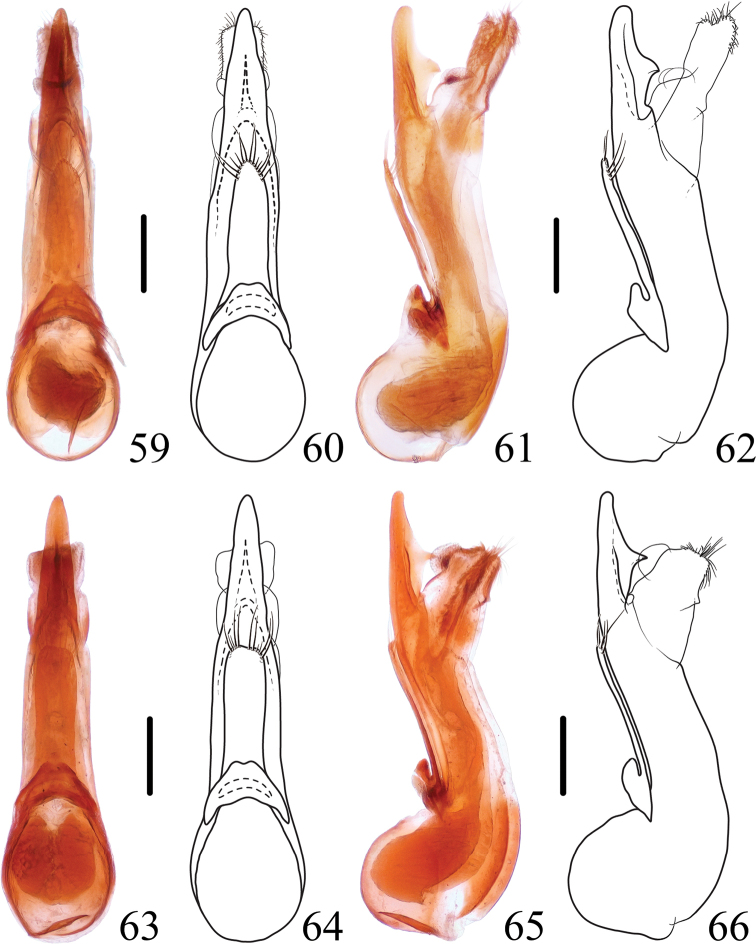
*Thoracostrongylusfujianensis***59–62** aedeagus from Guadun, ventral (**59, 60**) and lateral (**61, 62**) views **63–66** aedeagus from Guadun, ventral (**63, 64**) and lateral (**65, 66**) views. Scale bars: 0.2 mm.

#### Diagnosis.

The species shows some intraspecific variability (Figs 55–70) in the shape of the paramere and median lobe of the aedeagus. In general appearance, the species is similar to *T.acerosus*, *T.aduncatus*, and *T.diaoluoensis*, but can be keyed out by the aedeagal characters.

**Figures 67–73.**
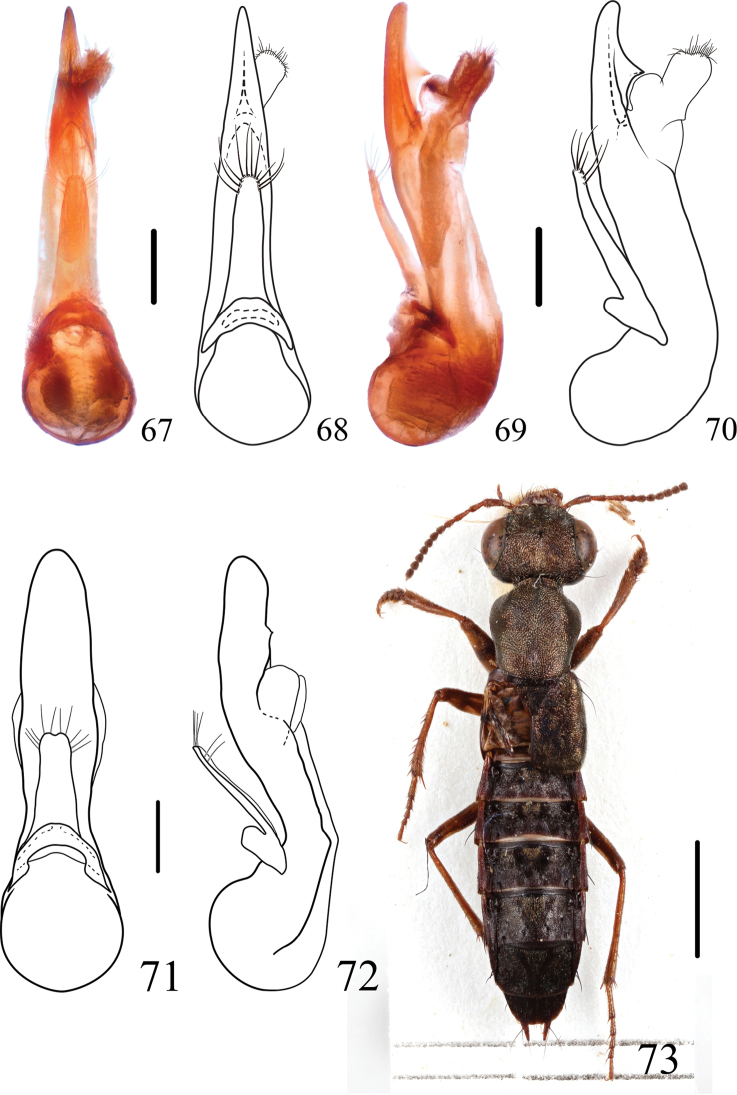
*Thoracostrongylus***67–70***T.fujianensis* aedeagus from Mt. Meihua, ventral (**67, 68**) and lateral (**69, 70**) views **71–72***T.malaisei* aedeagus, ventral (**71**) and lateral (**72**) views **73***T.miyakei* habitus. Scale bars: 0.2 mm (**67–72**); 2 mm (**73**).

#### Distribution.

China (Fujian).

### ﻿ Thoracostrongylus malaisei

Taxon classificationAnimaliaColeopteraStaphylinidae

Scheerpeltz, 1965

EF7DB307-14D6-57CA-A2E5-69D55ECC3B08

Figs 71 , 72 , 115 马来钝胸隐翅虫

Thoracostrongylus malaisei Scheerpeltz, 1965: 245; Yang et al. 2011: 428.

#### Material examined.

China – **Yunnan Prov.** • 1♀; 100 km W Baoshan, Gaoligongshan Nat. Res.; 14–21 June 1993; E. Jendek & O. Sausa leg.; NMW.

#### Distribution.

China (Yunnan), Myanmar.

#### Remarks.

The species was originally described from Myanmar, and was recorded from China by Yang, Zhou & Schillhammer in 2011 based on the one female mentioned above. However, there were some inconsistencies concerning morphometrics in that paper: The character used in the key “ratio of eye longitudinal diameter to temple length < 3”, applies only to the male. However, the measurements of female *T.malaisei* in the same paper were written as “CL: 0.98; PO: 0.28”, and the ratio of CL/PO should be 3.5 by calculation, which conflicts with the key. *Thoracostrongylusmalaisei* is most closely related to *T.brachypterus* sp. nov.; for diagnosis of these two species, see under the latter.

### ﻿ Thoracostrongylus miyakei

Taxon classificationAnimaliaColeopteraStaphylinidae

Bernhauer, 1943

F286F642-FCB7-558F-9602-735FFA2C9C79

Figs 73 , 116 三宅钝胸隐翅虫

Thoracostrongylus miyakei Bernhauer, 1943: 179; Yang et al. 2011: 428; Hu 2020: 349.

#### Material examined.

• 1♀; Taiwan, Taichung Hsien, Anmashan; alt. 2230 m; 30 April –4 May 1990; A. Smetana leg.; ASC.

#### Distribution.

China (Sichuan?, Taiwan).

#### Remarks.

The record for Sichuan reported by Yang et al. (2011) is doubtful: the record was published based on a specimen from Sichuan in coll. NMW. However, such a specimen does not exist, but there is a male (identified as *T.miyakei*) from Yunnan that was not mentioned in Yang et al. (2011). Numerous specimens from the mainland of east China have been examined in this paper and none of them is *T.miyakei*, creating a huge distributional gap between Sichuan and Taiwan. In addition, *T.miyakei* is a brachypterous species, making its occurrence in mainland China very unlikely. Since no male of that species from Taiwan was available for this paper, the solution to this problem must wait until males from Taiwan can be studied.

### ﻿ Thoracostrongylus sarawakensis

Taxon classificationAnimaliaColeopteraStaphylinidae

(Bernhauer, 1915)

AE766836-8FBB-57A3-BE0F-14905D2012F5

Fig. 117 沙捞越钝胸隐翅虫

Amichrotus sarawakensis Bernhauer, 1915h: 233.
Thoracostrongylus sarawakensis : Hammond 1984: 194, 195.
Ontholestes (Thoracostrongylus) doriae Gridelli, 1924: 207. Synonymized by de Rougemont 2016: 568.
Amichrotus doriae : Hammond 1984: 194, 195.

#### Material examined.

None.

#### Distribution.

China (Hainan?), Borneo.

#### Remarks.

The Chinese record of the species was published by de Rougemont (2016) without detailed locality data. The specimens in coll. Rougemont should be studied to confirm the occurrence of the species on Hainan.

### ﻿ Thoracostrongylus velutinus

Taxon classificationAnimaliaColeopteraStaphylinidae

Scheerpeltz, 1965

E4A102CF-D9FD-52BD-B3D8-BDDEC41467FF

**Figures 74–79.**
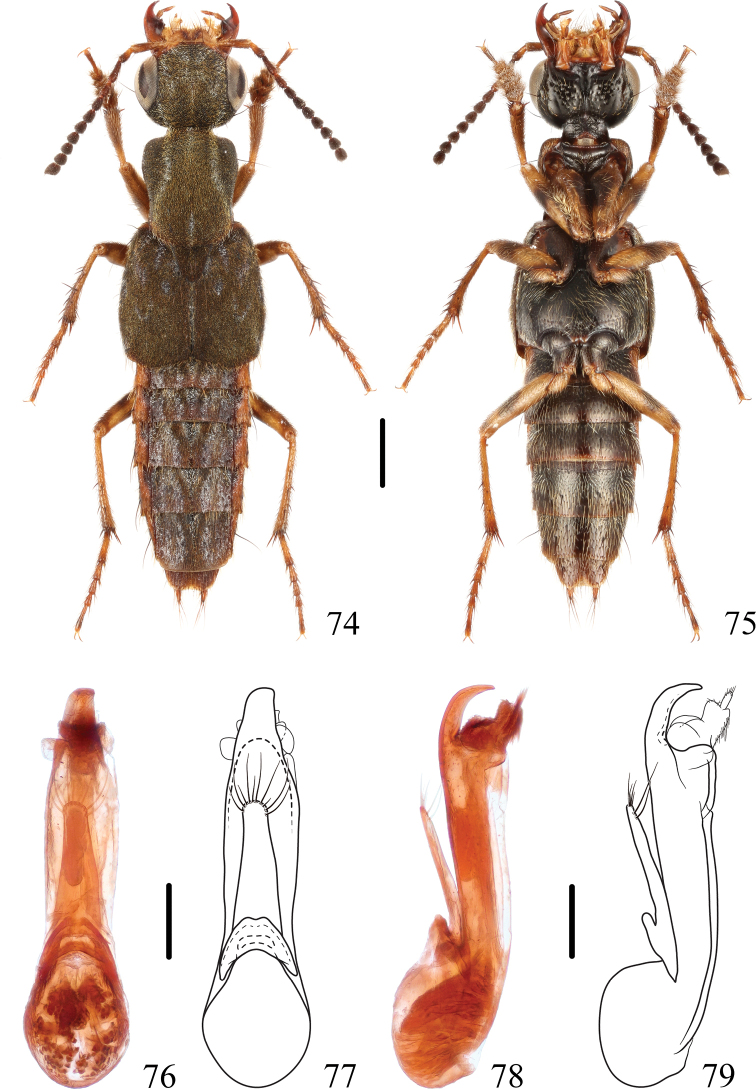
*Thoracostrongylusvelutinus***74, 75** habitus **76–79** aedeagus, ventral (**76, 77**) and lateral (**78, 79**) views. Scale bars: 1 mm (**74, 75**); 0.2 mm (**76–79**).

Figs 74–79 , 118 绒钝胸隐翅虫

Thoracostrongylus velutinus Scheerpeltz, 1965: 243; Yanget al. 2011: 430.

#### Material examined.

China – **Yunnan Prov.** • 1♂, 1♀; Gongshan County, Qiqi; alt. 2000 m; 29 June 2010; Wen-Xuan Bi leg.; SHNU • 3♀♀; Gongshan County, Qiqi; alt. 1900 m; 02 July 2010; Liang Tang leg.; SHNU • 1♂; Tengchong Coun., Baihualing; 24 May 2005; Hao Huang leg.; SHNU • 1♂; Tengchong County, Mingguang Town, Zizhi Vill; 25°42'N, 98°35'E; alt. 2300–2500 m; 30 April 2013; Song, Dai & Peng leg.; SHNU.

#### Measurements.

**Male**: BL: 7.3–8.9 mm, FL: 4.7–5.1 mm. HL: 1.22–1.33 mm, HW: 1.61–1.72 mm, CL: 0.83 mm, PO: 0.22–0.28 mm, PL: 1.67–1.78 mm, PW: 1.39–1.50 mm, EL: 2.11 mm, EW: 2.11–2.22 mm. HL/HW: 0.73–0.77, CL/PO: 3.00–3.75, PL/PW: 1.19–1.23, EL/EW: 0.95–1.00, HW/EW: 0.78–0.79, PW/EW: 0.66–0.68, HW/PW: 1.15–1.20. **Female**: BL: 8.4–10.3 mm, FL: 4.7–5.3 mm. HL: 1.28–1.45 mm, HW: 1.72–1.95 mm, CL: 0.89–0.95 mm, PO: 0.22–0.28 mm, PL: 1.67–1.89 mm, PW: 1.39–1.61 mm, EL: 2.00–2.34 mm, EW: 2.00–2.45 mm. HL/HW: 0.73–0.76, CL/PO: 3.20–4.00, PL/PW: 1.17–1.23, EL/EW: 0.95–1.00, HW/EW: 0.76–0.92, PW/EW: 0.63–0.69, HW/PW: 1.17–1.35.

#### Diagnosis.

The species can be easily distinguished from other species from southwest China by the abdominal tergites III–VI without a triangular, mediobasal, golden tomentose patch. In general appearance, *T.velutinus* is most similar to *T.formosanus*, but may be distinguished from the nominate ssp. of the latter by its smaller body size and paler mid and hind legs, and from *T.formosanusflavipes* by the dark antennae.

#### Distribution.

China (Yunnan), Myanmar.

### ﻿ Thoracostrongylus baishanzuensis sp. nov.

Taxon classificationAnimaliaColeopteraStaphylinidae

CAE66CC7-5A1D-5735-B124-107324CC4A5D

https://zoobank.org/2BCAB674-45E6-4A5B-BC62-53B506E0EC08

**Figures 80–85.**
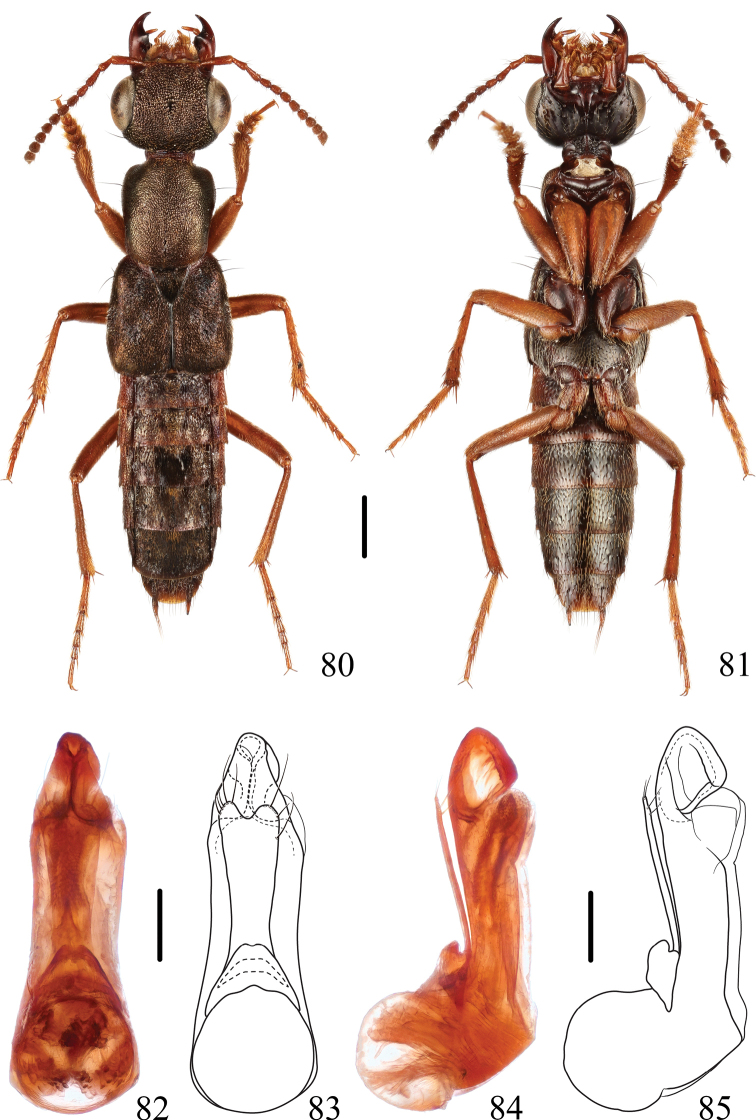
*Thoracostrongylusbaishanzuensis* sp. nov. **80, 81** habitus **82–85** aedeagus, ventral (**82, 83**) and lateral (**84, 85**) views. Scale bars: 1 mm (**80, 81**); 0.2 mm (**82–85**).

Figs 80–85 , 119 百山祖钝胸隐翅虫

#### Type material.

***Holotype.*** China – **Zhejiang Prov.** • ♂, glued on a card with labels as follows: “China: Zhejiang, Qingyuan, Baishanzu N.R.; 27°45'26"N, 119°12'08"E; alt. 1730 m; 02 May 2014; Peng, Song, Yan & Yu leg.” “Holotype / *Thoracostrongylus baishanzuensis* / Xia, Tang & Schillhammer” [red handwritten label]; SHNU. ***Paratypes.*** China – **Zhejiang Prov.** • 6♂♂, 2♀♀; same data as for the holotype; SHNU • 1♂, 1♀; Qingyuan, Baishanzu N.R.; 27°45'14"N, 119°11'55"E; alt. 1560–1750 m; 01 May 2014; Peng et al. leg.; SHNU • 1♂, 4♀♀; Lishui City, Qingyuan County, Baishanzu, Station to Peak; 27°45'20"N, 119°11'78"E; alt. 1721 m; 22 May 2015; Song & Yan leg.; SHNU • 1♂, 2♀♀; Lishui City, Qingyuan County, Baishanzu, Station to Peak; 27°45'20"N, 119°11'78"E; alt. 1721 m; 24 April 2015; Song & Yan leg.; SHNU • 1♀; Lishui City, Qingyuan County, Baishanzu; alt. 1500 m; 22–23 September 2008; Tang & Zhang leg.; SHNU.

#### Diagnosis.

The new species can be easily recognized by the combination of following characters: legs reddish yellow without dark markings, head slightly wider than or as wide as elytra, apical portion of median lobe of aedeagus (Figs 82–85) curved dorsad into a fin-shape, paramere bilobed.

#### Measurements.

**Male**: BL: 7.8–8.6 mm, FL: 4.3–4.9 mm. HL: 1.22–1.39 mm, HW: 1.72–1.95 mm, CL: 0.83–0.89 mm, PO: 0.28 mm, PL: 1.56–1.78 mm, PW: 1.33–1.50 mm, EL: 1.72–1.95 mm, EW: 1.72–1.95 mm. HL/HW: 0.71–0.74, CL/PO: 3.00–3.20, PL/PW: 1.15–1.23, EL/EW: 1.00, HW/EW: 1.00–1.03, PW/EW: 0.77–0.79, HW/PW: 1.26–1.31. **Female**: BL: 8.3–9.2 mm, FL: 4.7–5.2 mm. HL: 1.39–1.45 mm, HW: 1.95–2.06 mm, CL: 0.89–0.95 mm, PO: 0.28 mm, PL: 1.72–1.78 mm, PW: 1.45–1.61 mm, EL: 1.89–1.95 mm, EW: 1.89–2.00 mm. HL/HW: 0.68–0.71, CL/PO: 3.20–3.40, PL/PW: 1.10–1.19, EL/EW: 0.94–1.00, HW/EW: 1.00–1.03, PW/EW: 0.75–0.81, HW/PW: 1.28–1.35.

#### Description.

Forebody dark brown with a bronze tint, abdominal segments III and IV reddish brown, remaining segments gradually becoming darker apicad, labrum reddish brown, mandibles reddish brown with medial portions distinctly darker, maxillary and labial palpi reddish brown, antennae reddish brown, antennal club indistinctly darker, legs reddish brown without dark markings, elytra with few small patches of whitish pubescence, scutellum with black pubescence in apical half, abdominal tergites III–VII each with triangular mediobasal golden tomentose patch delimited by pair of dark tomentose spots, dark tomentose spots of tergites III and IV indistinct, dark tomentose spots of tergite V particularly large and dark, confluent apically, forming sagittate patch, dark tomentose spots of tergite VI similar to that of tergite V, but little smaller and distinctly lighter, dark tomentose spots of tergite VII indistinct.

Head slightly wider than or as wide as elytra; vertex with small longitudinal specular spot medially; surface densely covered with umbilicate punctures except specular median spot. Antennae with antennomere 1 longest, antennomeres 2 and 3 almost half as long as antennomere 1, antennomeres 4 and 5 longer than wide, antennomeres 6–10 gradually increasing in width and decreasing in length, antennomere 10 slightly longer than or as long as wide, antennomere 11 distinctly longer than wide, asymmetrical and subacuminate towards tip.

Pronotum widest behind anterior angles; punctation dense and umbilicate, very short and narrow impunctate midline in posterior quarter, pubescence golden, distinct on entire dorsal surface.

Elytra subquadrate, inconspicuously wider than long, slightly dilated posteriad; surface densely and finely, regularly punctate, with brassy pubescence, mixed with grey spots all over the disc. Scutellum triangular, finely and densely punctate, with black, velvety pubescence.

Abdomen with tergites densely punctate; tergites III–VII brown, tergite VII with apical palisade fringe.

**Male.** Sternite VIII with medio-apical emargination. Aedeagus (Figs 82–85) relatively stout, median lobe gradually narrowed apicad in apical fourth in ventral view, in lateral view, apical portion of median lobe curved dorsad forming distinct fin-shape; paramere very long, gradually widened apicad, apex bilobed, each lobe with five to six setae around apical margin.

**Female.** Sternite VIII with posterior margin entire.

#### Distribution.

China (Zhejiang).

#### Etymology.

This species is named after the type locality, Baishanzu, in Zhejiang Province, China.

### ﻿ Thoracostrongylus bicolor sp. nov.

Taxon classificationAnimaliaColeopteraStaphylinidae

3C224D22-3C4E-5379-A21E-23147A56B87A

https://zoobank.org/1F6192BE-17A1-4289-9684-83F45132333E

Figs 86–91 , 92–95 , 120 双色钝胸隐翅虫

#### Type material.

***Holotype.*** China – **Guangdong Prov.** • ♂, glued on a card with labels as follows: “China: Guangdong, Shaoguan, Ruyuan, Nanling N.R., Ruyang; 24°56'10"N, 113°00'18"E; alt. 1050–1200 m; 01–06 May 2021; Hu, Lin, Zhou & Li leg.” “Holotype / *Thoracostrongylus bicolor* / Xia, Tang & Schillhammer” [red handwritten label]; SHNU. ***Paratypes.*** China – **Guangdong Prov.** • 4♂♂, 1♀; same data as for the holotype; SHNU • 1♂, 3♀♀; Shaoguan, Ruyuan County, Nanling N.R., Ruyang; 24°55'49.5"N, 113°01'08"E; alt. 1000 m; 01 May 2021; Zhou & Li leg.; SHNU • 1♀; Ruyuan County, Nanling N.R., Qingshui Valley; 24°54'57"N, 113°01'55"E; alt. 900 m; 04 May 2015; Peng, Tu & Zhou leg.; SHNU. – **Hunan Prov.** • 1♀; Yizhang, Mangshan, Mengkengshi; 24°55'10"N, 112°58'37"E; alt. 1625 m; 26 August 2020; Zhong Peng leg.; SHNU. – **Guangxi Prov.** • 3♂♂, 2♀♀; Huanjiang, Jiuwan Mt., Yangmei’ao; 25°12'22.15"N, 108°40'32.01"E; alt. 1250 m; 25 April 2021; Tang, Peng, Cai & Song leg.; SHNU • 2♂♂; Xing’an County, Mao’er Shan; 25°53'07"N, 110°29'14"E; alt. 1143 m; 31 July 2014; Peng, Song, Yu & Yan leg.; SHNU • 1♀; Xing’an County, Mao’er Shan; 25°53'11"N, 110°28'13"E; alt. 810 m; 28 July 2014; Peng, Song, Yu & Yan leg.; SHNU • 1♂; Mt. Damingshan; 23°23'N, 103°29'E; alt. 1150–1250 m; 31 July 2012; Hu & Song leg.; SHNU • 1♀; Guilin City, Huaping N.R., Yunxi Valley; 25°34'00.62"N, 109°56'19.59"E; alt. 1460–1550 m; 23 April 2021; Yin, Zhang, Pan & Shen leg.; SHNU • 1♀; Jinxiu County, Mt. Shengtangshan; alt. 1200 m; 27 July 2011; Zhong Peng leg.; SHNU. – **Yunnan Prov.** • 5♀♀; NE Kunming; 25°08'40"N, 102°53'48"E; alt. 2290 m; 11 August 2014; mixed forest, sifted; V. Assing leg.; 3 VAC, 2 NMW • 5♀♀; NE Kunming; 25°08'35"N, 102°53'49"E; alt. 2320 m; 13 August 2014; mixed forest, sifted; V. Assing leg.; 4 VAC, 1 NMW • 1♀; Mt. W Xundian; 25°34'58"N, 103°08'42"E; alt. 2300 m; 15 August 2014; sifted; V. Assing leg.; VAC • 2♂♂, 2♀♀; Mt. W Xundian; 25°34'58"N, 103°08'42"E; alt. 2300 m; 16 August 2014; sifted; V. Assing leg.; 3 VAC, 1 NMW • 1 ex.; E Kunming, Xiaobailong Forest Park; 24°55'43"N, 103°05'22"E; alt. 2110 m; secondary pine forest, pine litter and litter at trail margin sifted; 10 August 2014; M. Schülke leg. [CH14-03]; MSC • 1♂, 2exs.; NE Kunming; 25°09'07"N, 102°53'46"E; alt. 2280 m; secondary pine forest, with scattered old alder, litter, sifted; 11 August 2014; M. Schülke leg. [CH14-04]; MSC • 1♂, 1 ex.; NE Kunming; 25°08'40"N, 102°53'48"E; alt. 2290 m; mixed deciduous forest with scattered pine trees, litter and mushrooms, sifted; 11 August 2014; M. Schülke leg. [CH14-05]; MSC • 1ex.; NE Kunming; 25°08'35"N, 102°53'49"E; alt. 2320 m; mixed forest with alder, oak, and pine, litter and mushrooms, sifted; 13 August 2014; M. Schülke leg. [CH14-06]; MSC • 1ex.; Mt. W Xundian; 25°34'58"N, 103°08'42"E; alt. 2300 m; mixed forest with alder, pine, shrub undergrowth, litter, twigs, and roots of herbs, sifted; 16 August 2014; M. Schülke leg. [CH14-09b]; MSC • 1♂; mountain W Yuxi; 24°27'11"N, 102°29'58"E; alt. 2250 m; secondary mixed forest, litter, roots, and moss sifted; 31 August 2014; M. Schülke leg. [CH14-23]; MSC.

**Figures 86–91.**
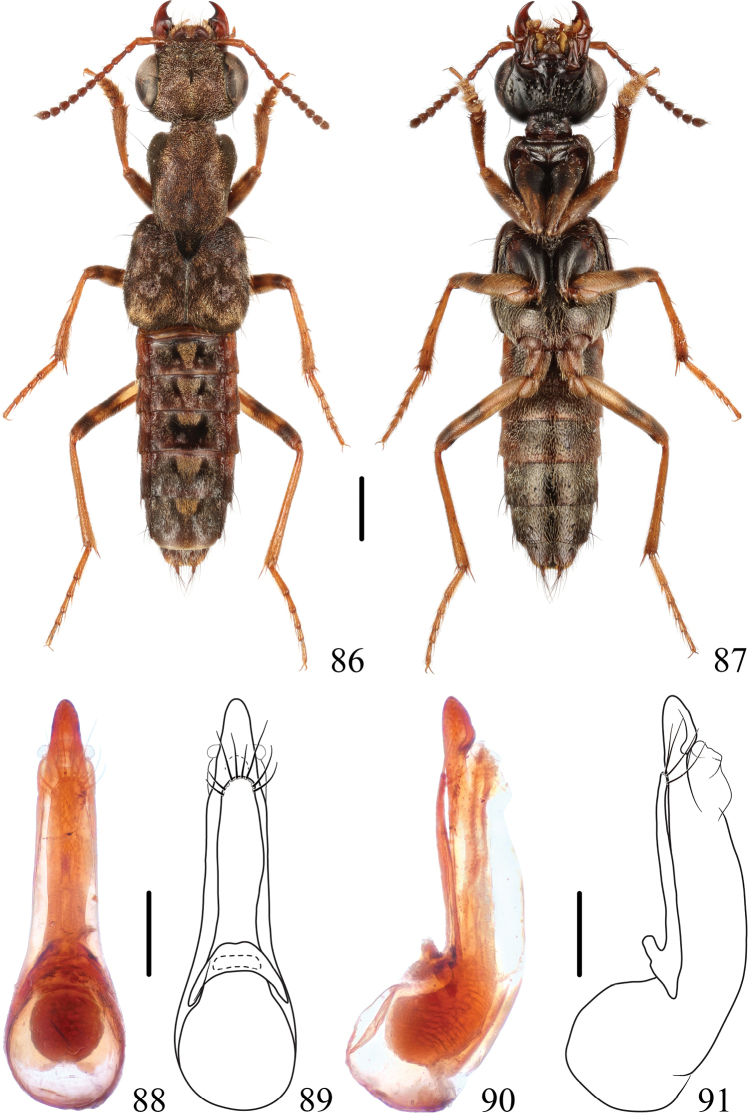
*Thoracostrongylusbicolor* sp. nov. **86, 87** habitus **88–91** aedeagus, ventral (**88, 89**) and lateral (**90, 91**) views. Scale bars: 1 mm (**86, 87**); 0.2 mm (**88–91**).

#### Measurements.

**Male**: BL: 8.1–10.1 mm, FL: 4.5–4.9 mm. HL: 1.22–1.39 mm, HW: 1.78–1.95 mm, CL: 0.83–0.95 mm, PO: 0.22–0.28 mm, PL: 1.67–1.78 mm, PW: 1.39–1.45 mm, EL: 1.78–2.06 mm, EW: 1.95–2.09 mm. HL/HW: 0.69–0.74, CL/PO: 3.20–4.25, PL/PW: 1.15–1.24, EL/EW: 0.91–1.00, HW/EW: 0.91–0.97, PW/EW: 0.69–0.74, HW/PW: 1.23–1.35. **Female**: BL: 7.9–11.0 mm, FL: 4.6–5.4 mm. HL: 1.22–1.50 mm, HW: 1.83–2.11 mm, CL: 0.89–1.06 mm, PO: 0.22–0.28 mm, PL: 1.67–1.89 mm, PW: 1.39–1.61 mm, EL: 1.95–2.22 mm, EW: 1.95–2.28 mm. HL/HW: 0.67–0.74, CL/PO: 3.40–4.75, PL/PW: 1.11–1.26, EL/EW: 0.97–1.00, HW/EW: 0.93–0.97, PW/EW: 0.66–0.73, HW/PW: 1.28–1.41.

#### Diagnosis.

The new species is similar to *T.baishanzuensis* sp. nov., but it can be easily recognized from latter by the bicolored femora. From other species of east and southeast China, it can be easily recognized by the bicolored abdomen.

#### Description.

The new species is similar to *T.baishanzuensis* sp. nov. in most aspects except for the following characters: abdominal tergites III–VII each with a longer and more distinct triangular, mediobasal, golden tomentose patch; femora each with median dark mark and apical dark mark, although the apical dark markings of the forelegs are less distinct.

**Male.** Sternite VIII with medioapical emargination. Aedeagus (Figs 88–95) slender, median lobe gradually narrowed apicad with round apex in ventral view, apex of median lobe expanded dorsad in lateral view; paramere relatively long, apex wide and round with approximately 11 setae around the apical margin.

**Figures 92–95.**
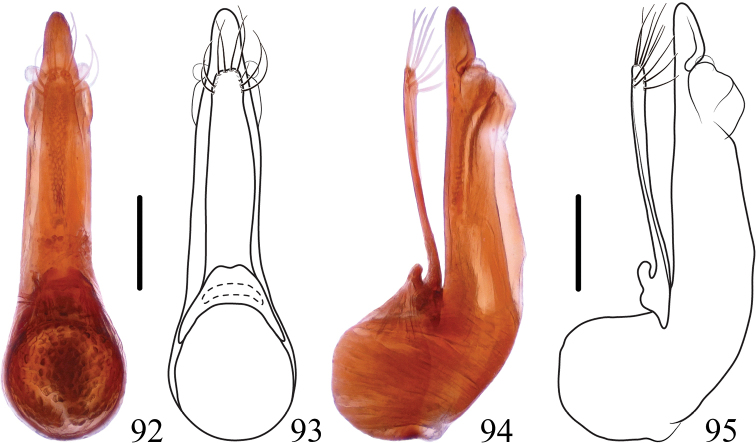
*Thoracostrongylusbicolor* sp. nov. **92–95** aedeagus from Guangxi, ventral (**92, 93**) and lateral (**94, 95**) views. Scale bars: 0.2 mm.

**Female.** Sternite VIII with posterior margin entire.

#### Distribution.

China (Guangdong, Hunan, Guangxi, and Yunnan).

#### Etymology.

This species is named after its bicolored abdomen.

### ﻿ Thoracostrongylus brachypterus sp. nov.

Taxon classificationAnimaliaColeopteraStaphylinidae

64AD2C23-8CAF-5F76-AC76-9219E05B01D8

https://zoobank.org/09964804-5ECF-43BA-817C-6F2A046D2759

**Figures 96–101.**
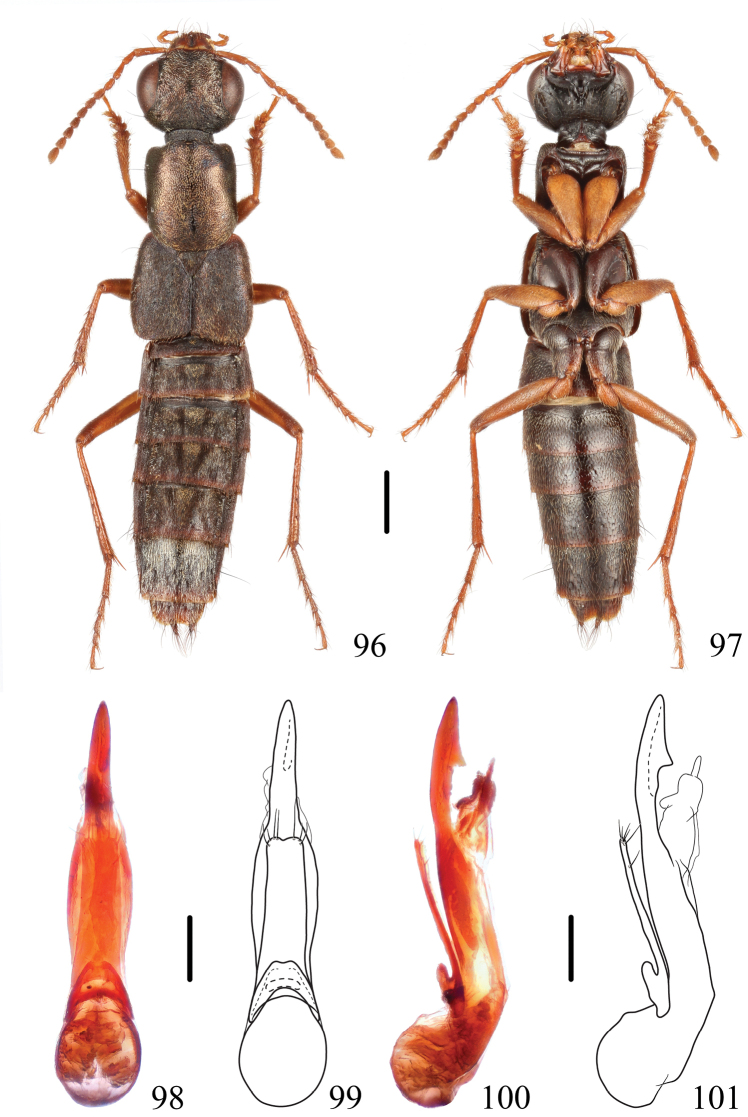
*Thoracostrongylusbrachypterus* sp. nov. **96, 97** habitus **98–101** aedeagus, ventral (**98, 99**) and lateral (**100, 101**) views. Scale bars: 1 mm (**96, 97**); 0.2 mm (**98–101**).

Figs 96–101 , 121 短翅钝胸隐翅虫

#### Type material.

***Holotype.*** China – **Sichuan Prov.** •♂, glued on a card with labels as follows: “China: Sichuan, Muli Tibetan Autonomous County, Mianbu Yakou; 27°68'N, 101°22'E; alt. 3100 m; 04 June 2012; Hao Huang. leg.” “Holotype / *Thoracostrongylus brachypterus * / Xia, Tang & Schillhammer” [red handwritten label]; SHNU. ***Paratypes.*** China – **Sichuan Prov.** • 1♀; S Sichuan, pass 20km S MULI (BOWA); 27.45°N, 101.13°E; 28–29 June 1998; mixed forest cca. 3500m; Jaroslav Turna leg.; NMW • 21♂♂, 9♀♀; S-Sichuan, pass betw. Yanyuan/Muli; alt. 3244 m; 27.68638°N, 101.22335°E; 11–18 June 2017; C. Reuter leg.; 20 BFC, 10 NMW • 1♂; S-Sichuan, pass ~ 50km NE Yanyuan to Xichang; alt. 2950 m, 27°33'11"N, 101°45'04"E; 07–18 June 2017; C. Reuter leg.; BFC.

#### Measurements.

**Male**: BL: 7.6–9.3 mm, FL: 4.1–4.6 mm. HL: 1.20–1.30 mm, HW: 1.50–1.70 mm, CL: 0.80–0.89 mm, PO: 0.22–0.25 mm, PL: 1.55–1.78 mm, PW: 1.23–1.45 mm, EL: 1.60–1.72 mm, EW: 1.70–1.89 mm. HL/HW: 0.77–0.80, CL/PO: 3.23–4.00, PL/PW: 1.23–1.26, EL/EW: 0.91–0.94, HW/EW: 0.88–0.90. **Female**: BL: 11.0 mm, FL: 4.8–5.0 mm. HL: 1.35–1.40 mm, HW: 1.80–1.85 mm, CL: 0.80–0.90 mm, PO: 0.30–0.35 mm, PL: 1.75–1.80 mm, PW: 1.55 mm, EL: 1.85 mm, EW: 1.95 mm. HL/HW: 0.73–0.78, CL/PO: 2.33–3.03, PL/PW: 1.13–1.16, EL/EW: 0.95, HW/EW: 0.92–0.95.

#### Diagnosis.

The new species is the only brachypterous species of the genus so far that is known from mainland China, except for a potential record of a brachypterous *T.malaisei*, from which it can be separated as indicated above. The *T.malaisei* specimens from the type locality have rather short elytra and developed hindwings, which may be functional or not since the palisade fringe on tergite VII is very narrow. *Thoracostrongylusmiyakei* from Taiwan also has weakly developed, non-functional hind wings and no palisade fringe on tergite VII, which differs from the new species by pronotum without impunctate midline.

#### Description.

The new species is almost identical to *T.malaisei*, from which it differs, in addition to the different aedeagus, by the differently colored labrum, which is reddish with each lobe with a large, dark brown, central spot (in *T.malaisei* with a black medial margin along medial excision). Most specimens of *T.malaisei* have at least a very narrow palisade fringe on tergite VII, which is lacking only in the single specimen from Yunnan.

**Male.** Sternite VIII with medioapical emargination. Aedeagus (Figs 98–101) slender and long, median lobe swollen in middle third and then narrowed apicad in ventral view; in lateral view, median lobe with subapical tooth on dorsal side in apical sixth; paramere rather wide, subparallel-sided, apical margin with slight medial notch, with approximately seven setae.

**Female.** Sternite VIII with posterior margin entire.

#### Distribution.

China (Sichuan).

#### Etymology.

This specific name (derived from Greek) means “short winged”.

### ﻿ Thoracostrongylus chrysites sp. nov.

Taxon classificationAnimaliaColeopteraStaphylinidae

ABFA590D-A8A1-5798-BDDA-466161550D41

https://zoobank.org/8DE3842B-DDF8-4AE8-85AE-9E149BDC418B

**Figures 102–107.**
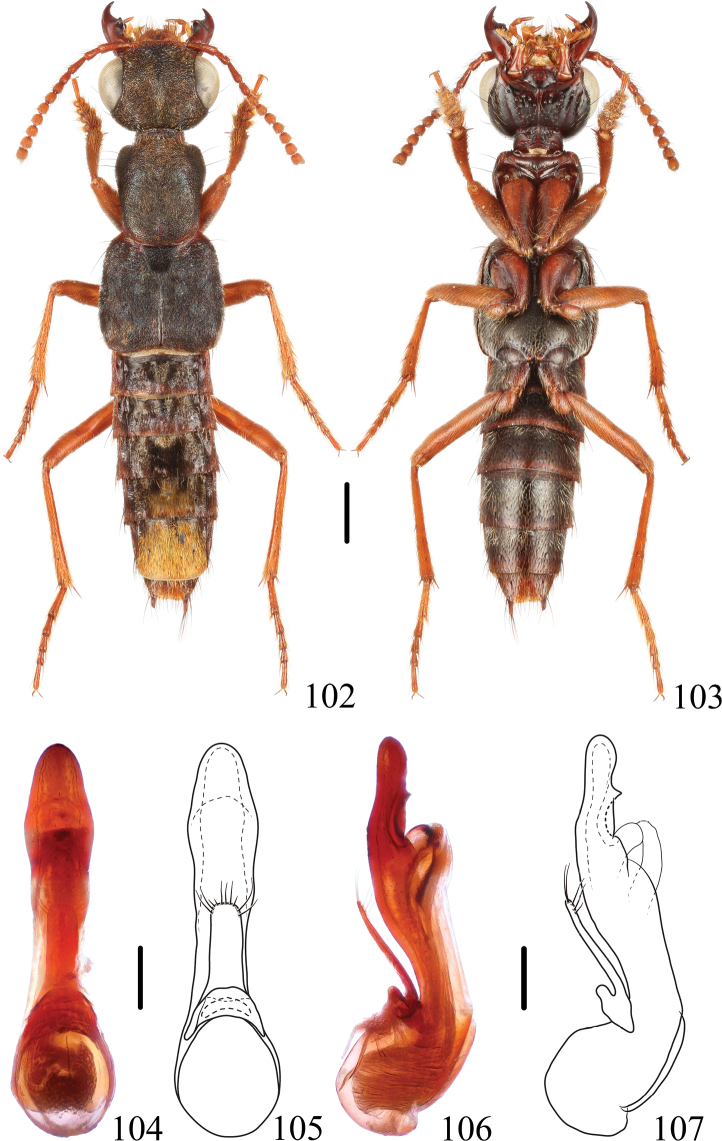
*Thoracostrongyluschrysites* sp. nov. **102, 103** habitus **104–107** aedeagus, ventral (**104, 105**) and lateral (**106, 107**) views. Scale bars: 1 mm (**102, 103**); 0.2 mm (**104–107**).

Figs 102–107 , 122 金斑钝胸隐翅虫

#### Type material.

***Holotype.*** China – **Fujian Prov.** •♂, glued on a card with labels as follows: “China: Fujian, Wuyishan City, Guadun Vill.; 27°45'N, 117°38'E; alt. 1800 m; 01 June 2012; Peng & Dai leg.” “Holotype / *Thoracostrongylus chrysites* / Xia, Tang & Schillhammer” [red handwritten label]; SHNU. ***Paratypes.*** China – **Fujian Prov.** • 3♀♀; same data as for the holotype; SHNU • 1♀; Wuyishan City, Guadun Vill.; 27°44'N, 117°38'E; alt. 1700–1800 m; 31 May 2012; Peng & Dai leg.; SHNU.

#### Measurements.

**Male**: BL: 9.0 mm, FL: 5.0 mm. HL: 1.45 mm, HW: 1.95 mm, CL: 0.95 mm, PO: 0.28 mm, PL: 1.67 mm, PW: 1.45 mm, EL: 1.95 mm, EW: 2.00 mm. HL/HW: 0.74, CL/PO: 3.40, PL/PW: 1.15, EL/EW: 0.97, HW/EW: 0.97, PW/EW: 0.72, HW/PW: 1.35. **Female**: BL: 9.5–10.0 mm, FL: 4.7–5.1 mm. HL: 1.39–1.50 mm, HW: 1.89–2.06 mm, CL: 0.83–1.00 mm, PO: 0.22–0.33 mm, PL: 1.67–1.78 mm, PW: 1.45–1.56 mm, EL: 1.95–2.11 mm, EW: 1.95–2.11 mm. HL/HW: 0.73–0.74, CL/PO: 2.50–4.25, PL/PW: 1.14–1.19, EL/EW: 0.97–1.00, HW/EW: 0.97, PW/EW: 0.72–0.74, HW/PW: 1.31–1.35.

**Figures 108–122.**
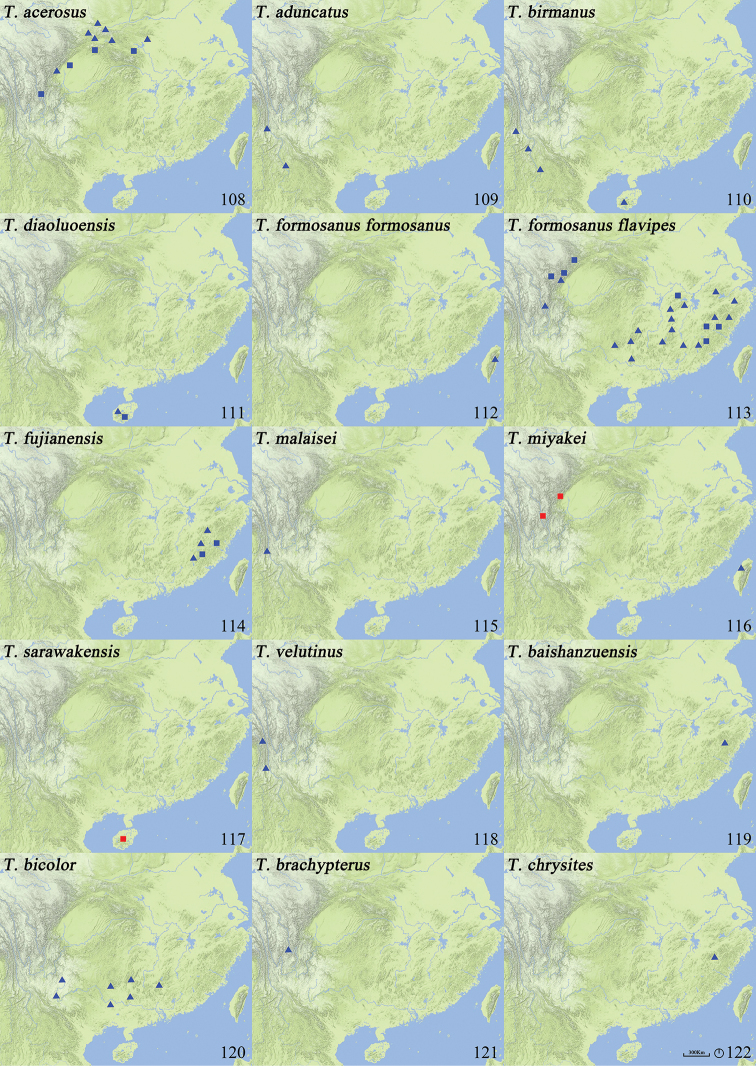
Distribution map of *Thoracostrongylus* species of China **108***T.acerosus***109***T.aduncatus***110***T.birmanus***111***T.diaoluoensis***112***T.formosanusformosanus***113***T.formosanusflavipes***114***T.fujianensis***115***T.malaisei***116***T.miyakei***117***T.sarawakensis***118***T.velutinus***119***T.baishanzuensis***120***T.bicolor***121***T.brachypterus***122***T.chrysites*. Triangle, localities of specimens examined in this paper; square, localities of specimens listed in previous papers. Red, doubtful localities; blue, trustede localities.

#### Diagnosis.

The new species can be easily recognized by the reddish yellow femora and abdominal tergite VII fully covered with golden pubescence.

#### Description.

The new species is similar to *T.baishanzuensis* sp. nov. except for the following characters: pronotum reddish along posterior margin, elytra reddish at base, abdominal segments with posterior margin reddish, legs reddish yellow without dark markings, although indistinct dark markings may be present near base of profemora; abdominal tergite VI with larger median golden tomentose patch, reaching posterior margin of tergite, pair of dark tomentose spots very small; abdominal tergite VII completely covered with golden pubescence.

**Male.** Sternite VIII with medioapical emargination. Aedeagus (Figs 104–107) slender, in ventral view, median lobe slightly widened in apical fifth, apex broadly rounded; in lateral view, median lobe with subapical dorsal tooth, apex rounded.

**Female.** Sternite VIII with posterior margin entire.

#### Distribution.

China (Fujian).

#### Etymology.

The species is named after the golden pubescence of abdominal tergite VII.

### ﻿Key to Chinese species of *Thoracostrongylus*

**Table:** 

1	Last three antennomeres whitish, distinctly lighter than previous antennomeres. China (Hainan?), Borneo	***T.sarawakensis***
–	Last three antennomeres not whitish, similar to or slightly darker than previous antennomeres	**2**
2	Posterior margin of abdominal tergite VII without palisade fringe; hindwings reduced	**3**
–	Posterior margin of abdominal tergite VII with more or less distinct palisade fringe; macropterous	**5**
3	Labrum reddish, each lobe with margin along median excision blackish. Specimens of *T.malaisei* without palisade fringe at posterior margin of tergite VII	**6**
–	Labrum reddish, each lobe with variably large dark brown spot in center	**4**
4	Pronotum usually with an almost complete, but narrow impunctate midline, rarely with only a specular medio-longitudinal patch in posterior half; China (Sichuan)	***T.brachypterus***
–	Pronotum without impunctate midline; China (Sichuan?, Taiwan)	***T.miyakei***
5	Abdominal tergites III–VI without triangular mediobasal golden tomentose patch, instead with some silvery pubescence	**6**
–	Abdominal tergites III–VI each with triangular mediobasal golden tomentose patch	**9**
6	Fore body with coppery hue, interstices of punctures slightly wider, thus more shiny; palisade fringe at posterior margin of tergite VII very narrow or lacking. China (Yunnan), Myanmar	***T.malaisei***
–	Fore body with olive greenish hue, punctation extremely dense, fore body thus very matt; palisade fringe at posterior margin of tergite VII distinct	**7**
7	Antennae and legs (except for band on femora) entirely reddish. China (Zhejiang, Fujian, Hubei, Hunan, Sichuan, Guangxi, Guangdong, Anhui, Jiangxi)	***T.formosanusflavipes***
–	Antennae with variable number of segments (at least distal six) dark brown or black	**8**
8	Meso- and metatibiae and -tarsi usually black. More robust build. China (Taiwan)	***T.formosanusformosanus***
–	Meso- and metatibiae and -tarsi reddish. Body smaller. China (Yunnan), Myanmar	***T.velutinus***
9	Femora reddish yellow without black markings	**10**
–	Femora reddish yellow with black markings	**11**
10	Head narrower than elytra in most specimens; abdominal tergite VII fully covered with golden pubescence. China (Fujian)	***T.chrysites***
–	Head as wide as or slightly wider than elytra; abdominal tergite VII with triangular mediobasal golden tomentose patch. China (Zhejiang)	***T.baishanzuensis***
11	Abdominal sternites with long and dense pubescence; posterior margin of male 8^th^ sternite deeply emarginate (Fig. 39). China (Yunnan, Hainan), India, Myanmar	***T.birmanus***
–	Abdominal sternites with relatively short and sparse pubescence; posterior margin of male 8^th^ sternite shallowly emarginate	**12**
12	Abdominal sternites III–V reddish brown, lighter than remaining sternites. China (Guangdong, Hunan, Guangxi, Yunnan)	***T.bicolor***
–	Abdominal sternites III–V brown, similar to remaining sternites; four species that can be separated with certainty only by the shape of the aedeagus	**13**
13	In lateral view, median lobe of aedeagus with an apical or subapical tooth on dorsal side	**14**
–	In lateral view, median lobe of aedeagus without an apical or subapical tooth on dorsal side (Figs 42–45). China (Hainan)	***T.diaoluoensis***
14	In lateral view, apex of median lobe of aedeagus pointing dorsad, forming a subapical tooth (Figs 17–32). China (Yunnan)	***T.aduncatus***
–	In lateral view, median lobe of aedeagus without distinct subapical tooth	**15**
15	In ventral view, apex of median lobe of aedeagus with a sharp tip (Figs 3–14). China (Hubei, Sichuan, Shaanxi, Gansu, Henan)	***T.acerosus***
–	In ventral view, apex of median lobe of aedeagus with a blunt tip (Figs 55–70). China (Fujian)	***T.fujianensis***

## Supplementary Material

XML Treatment for Thoracostrongylus acerosus

XML Treatment for Thoracostrongylus aduncatus

XML Treatment for Thoracostrongylus birmanus

XML Treatment for Thoracostrongylus diaoluoensis

XML Treatment for Thoracostrongylus formosanus formosanus

XML Treatment for Thoracostrongylus formosanus flavipes

XML Treatment for Thoracostrongylus fujianensis

XML Treatment for Thoracostrongylus malaisei

XML Treatment for Thoracostrongylus miyakei

XML Treatment for Thoracostrongylus sarawakensis

XML Treatment for Thoracostrongylus velutinus

XML Treatment for Thoracostrongylus baishanzuensis

XML Treatment for Thoracostrongylus bicolor

XML Treatment for Thoracostrongylus brachypterus

XML Treatment for Thoracostrongylus chrysites
